# Transcription factors organize into functional groups on the linear genome and in 3D chromatin

**DOI:** 10.1016/j.heliyon.2023.e18211

**Published:** 2023-07-17

**Authors:** Rakesh Netha Vadnala, Sridhar Hannenhalli, Leelavati Narlikar, Rahul Siddharthan

**Affiliations:** a The Institute of Mathematical Sciences, Chennai, India; b Homi Bhabha National Institute, Mumbai, India; c National Cancer Institute, National Institutes of Health, Bethesda, MD, USA; d Department of Data Science, Indian Institute of Science Education and Research, Pune, India

**Keywords:** Chromatin, Transcription factors

## Abstract

Transcription factors (TFs) and their binding sites have evolved to interact cooperatively or competitively with each other. Here we examine in detail, across multiple cell lines, such cooperation or competition among TFs both in sequential and spatial proximity (using chromatin conformation capture assays), considering in vivo binding data as well as TF binding motifs in DNA. We ascertain significantly co-occurring (“attractive”) or avoiding (“repulsive”) TF pairs using robust randomized models that retain the essential characteristics of the experimental data. Across human cell lines TFs organize into two groups, with intra-group attraction and inter-group repulsion. This is true for both sequential and spatial proximity, and for both in vivo binding and sequence motifs. Attractive TF pairs exhibit significantly more physical interactions suggesting an underlying mechanism. The two TF groups differ significantly in their genomic and network properties, as well in their function—while one group regulates housekeeping function, the other potentially regulates lineage-specific functions, that are disrupted in cancer. Weaker binding sites tend to occur in spatially interacting regions of the genome. Our results suggest that a complex pattern of spatial cooperativity of TFs and chromatin has evolved with the genome to support housekeeping and lineage-specific functions.

## Highlights

• We present a method for learning significantly co-occurring pairs of TFs from high-throughput experiments.
• TFs associate in two groups, differing in protein-protein interactions, protein-DNA binding, and biological function.
• One group tends to bind proximally to genes, and is conserved in function across cell-types.
• The other tends to bind distally, and its function is more specific to cell lineage.
• Cooperativity among TFs appears modulated by 3D chromatin architecture as well as TF affinity to specific DNA binding sites.

## Graphical abstract

**Figure:**
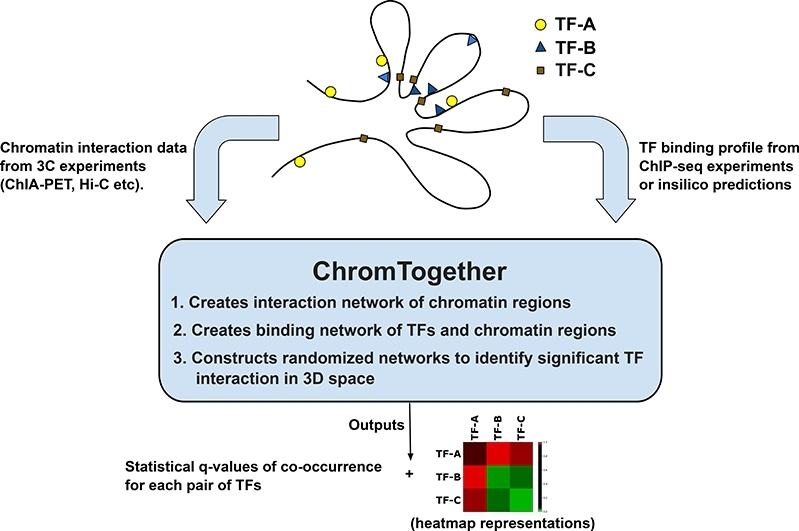

## 1 Introduction

DNA-binding proteins play a key role in nuclear and cellular processes. In particular, RNA polymerase transcribes genes to RNA molecules; transcription factors (TFs) help regulate transcription by binding to specific sites in DNA; histones organize DNA into a three-dimensional chromatin structure; and the cohesin complex and the CTCF factor further control this 3D organization. Gene expression is controlled by the complicated combinatorial interplay of accessibility of DNA, remodelling of chromatin, binding of proteins such as TFs to DNA, recruitment of RNA polymerase, and the spatial interaction of all these factors on a three-dimensional stage.

All these aspects can be probed today by high-throughput techniques: Hi-C and variants can detect interacting regions in chromatin [1], [2], [3], [4], [5]; ChIP-seq and related techniques can assay binding sites for proteins in vivo genome-wide [6]; and open chromatin can be ascertained by techniques such as DNase-seq and ATAC-seq [7], [8].

Transcriptional regulation has traditionally been studied by considering DNA as a linear sequence containing genes, promoters, enhancers and TF-binding sequence motifs [9], [10], [11], [12], [13]. Only recently have attempts been made to integrate multiple sources of data to form a three-dimensional understanding of transcriptional regulation. Malin et al. (2015) [14] showed that spatially clustered enhancers containing low affinity homotypic TF motif sites exhibit greater in vivo TF occupancy than the motif sites not appearing in clusters, highlighting the role of spatial enhancer cluster in potentially assisting binding at weaker TF binding sites. Ma et al. (2018) [15] studied co-localization of homotypic and heterotypic motif sites in Hi-C contact regions, suggesting the existence of a spatial TF interaction network. Others have considered 3D chromatin in the context of protein-protein interaction (PPI) networks and pathways [16], [17], [18].

Here, using chromatin interaction (ChIA-PET) and ChIP-seq data in multiple cell lines, as well as motif information, we explore the interplay between TFs in three-dimensional chromatin. Specifically, we examine the co-binding of pairs of TFs in spatially proximal regions and assess whether such co-binding occurs significantly more or less often than one would expect by chance. This is accomplished with a careful randomization technique that respects essential properties of the interaction network.

In the cell lines examined here, we find that almost all pairs of TFs tend to co-occur either significantly more often (which we call “attraction”), or significantly less often (which we call “repulsion”) than by chance. For a given cell line, we find that the pattern of attraction or repulsion agrees with what is seen in sequentially-adjacent regions. Interestingly, a similar pattern emerges if one uses only motif information, i.e. no experimental binding data. These two groups appear functionally different, in their location of binding relative to transcriptional start sites (TSS), in their protein-protein-interaction (PPI) network properties, and in the functional nature of their target genes.

We provide a tool to perform similar analyses on chromatin interaction data (such as Hi-C or ChIA-PET) and ChIP-seq (or motif) datasets, ChromTogether available at https://github.com/rakeshnetha14/ChromTogether. In the future we hope to use such information to improve the performance of motif-finding and in-silico TFBS prediction.

We should note that while some of the DNA-binding proteins in our ChIP-seq data, such as cohesin subunits, are not transcription factors, for conciseness we refer to all assayed DNA-binding proteins as TFs.

## 2 Materials and methods

### 2.1 Interaction network and binding network

High-throughput chromatin conformation capture-based experiments (such as Hi-C, ChIA-pet, capture Hi-C) report an interaction score between multiple pairs of regions in the genome. Here, we use various ChIA-pet datasets and one Hi-C dataset. In each case, after merging regions that occur within 2 kbp of each other and filtering out pairs of interactions within 2 kbp of each other, we obtain a set of long-range interaction pairs. We represent this data as an undirected graph, with a node denoting a region and an edge denoting a long-range interaction. We remove regions with an unrealistically high degree (>20) or length (>30 kbp) as these are likely the result of the merging step or from the experiment reporting a cell population average. We call this the “interaction network” arising from that experiment.

**Figure 1:**
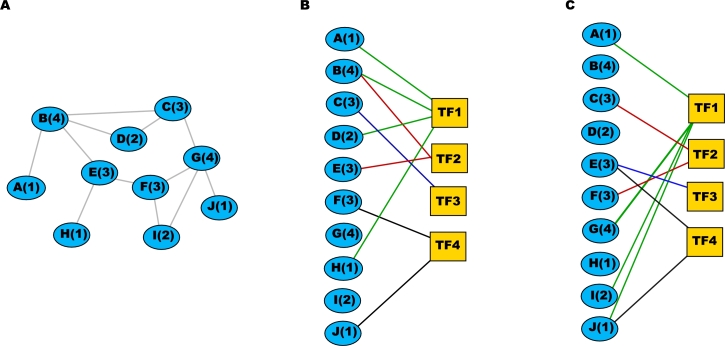
(A) The interaction network, where each node is a contiguous genomic region and links indicate contacts between regions as determined by chromatin interaction data. The degree of each region in the region-region interaction network is shown in brackets. (B) The binding network, a bipartite network where blue nodes are regions as in (A), yellow squares are TFs, and links indicate binding of a TF to a region. Links from TF1 are shown in green, from TF2 in red, for clarity. (C) A possible randomization of links where each TF-region link is randomly reassigned from the TF to another (possibly the same) region such that the region-region interaction degree of the bound region, in brackets, is approximately preserved.

We next look at TF-binding information from ChIP-seq data for the same cell-type. We construct a corresponding “binding network” which contains all region nodes from the interaction network as well as additional TF nodes. An edge in this network connects a TF node to a region node, if there is evidence of the TF binding to that region in a ChIP-seq experiment. The interaction network and binding network are illustrated in Fig. 1A and 1B.

The goal is to assess TF pairs that co-occur via the interaction network. For this purpose, all instances of TFs *i* and *j* binding to adjacent nodes in the interaction network are considered as co-occurrences of the two TFs. We estimate the significance of these co-occurrences by simulating random networks whose construction is described below. Defining co-occurrence that is significantly more frequent than random as “attraction” and co-occurrence that is significantly less frequent than random as “repulsion”, we ask whether there is any biological significance to attracting or repelling pairs; whether such pairs are conserved across cell types and across species; whether such attracting (repelling) pairs also attract (repel) within short (500–2000 bp) contiguous regulatory sequence (e.g., promoters or enhancers); and whether, if one looks purely at sequence motifs rather than ChIP-seq binding events, similar patterns are seen.

In supplementary material (A.1) we provide some statistics of the interaction network. In particular, most nodes in the Pol-II ChIA-PET network have a degree ≤ 5; and most have a genomic length of < 1000 bp.

### 2.2 Construction of randomized networks

Our randomized networks are constructed to preserve essential properties of the original data: in particular, the coordination numbers of TF nodes in the binding network and of region nodes in the interaction network. This is to minimize bias arising from the fact that some TFs exhibit higher connectivity and more widespread binding than others.

We view the binding network as a bipartite network, with nodes representing regions in the interaction network linked to nodes representing TFs in the ChIP-seq data. The region-region interaction degree of each region is known and the node corresponding to each region is labelled, in the binding network, with its region-region interaction degree.

We generate random networks by reassigning these TF-region links with the constraint that each TF-region link is assigned from the same TF to a region with a similar region-region degree. Specifically: we reassign a TF link to a region *x* of degree ≤ 5 to a random region *y* with exactly the same region-region degree as region *x*; a region *x* with degree between 6 and 10 to a random region *y* with degree within ± 2 of region *x*'s degree; and a region *x* with degree >10 to a random region of degree >10. This is motivated by the degree distribution of the interaction network depicted in supplementary Fig. A.1(A) This ensures that in the randomized bipartite network, each TF node has the same out-degree as originally, and that the interaction-network degree distribution for TF target regions is approximately conserved. This is illustrated in Fig. 1 (C). The supplementary material Fig. A.2 shows this conservation for each TF-region in the original network throughout the 1000 randomization steps.

### 2.3 Significance assessment: *q*-values

We construct 1000 such randomized networks. From these, we calculate two *p*-values for each pair of TFs: $p_{ij}$ is the *p*-value for TFs *i* and *j* co-occurring equally or more often than in the real data under the null that they co-occur randomly, calculated as the fraction of random networks where this happens; and similarly $p_{ij}^{\prime }$ is the *p*-value for *i* and *j* co-occurring equally or *less* often than in the real data. To correct for multiple pairwise comparisons, we use the full list of *p*-values to calculate *q*-values *q* and $q^{\prime }$, which indicate false discovery rates, following the Benjamini-Hochberg procedure [19]. Small values of *q*, and $q^{\prime }$, respectively indicate TF pairs co-occurring significantly more often, and less often, than chance. Finally, we show the significant *q* and $q^{\prime }$ values (less than 0.05) on a heatmap, in green and red respectively. In order to show both attraction and repulsion on the same heatmap, we plot *q* in green and ${\overset{˜}{q}}_{ij}=1-q_{ij}^{\prime }$, ${\overset{˜}{q}}_{ij}\geq 0.95$ in red (with the brightest reds being the largest, i.e. most significant values). As a control, we selected one of the random network as real network and compared it with all other random networks and results in no significant attraction or repulsion TF pairs.

### 2.4 Motif analysis

We predict motif instances with FIMO [20] with the default parameters, using TF motifs from the JASPAR database [21] bundled with the MEME suite [22] (JASPAR2018_CORE_vertebrates_redundant.meme). Rather than use all 719 motifs in that file, we identified similar motifs using TOMTOM [23] and clustered them into 93 groups, in each of which we picked the most informative motif as a representative, using the motif information score $I=\sum_{n}\left(2+\sum_{b=A,C,G,T}W_{nb}\log_{2}W_{nb}\right)$ where $W_{nb}$ is the PWM probability for position *n* and nucleotide *b*. The selected TFs and their motif PWM ids are given in supplementary material Table A.3).

In the section “Motif strength correlates with spatial interaction” we use the log likelihood ratio of a site as its “motif score”. Given a sequence *S* of length *L* and a PWM *W* of the same length, the LLR = log⁡(P(S|W)/P(S|B)) $=\sum_{n=1}^{L}\log W_{nS_{n}}-\log B_{S_{n}}$ where $S_{n}$ denotes the nucleotide at the *n*th position of *S*, and *B* is a background model for the sequence (probability 0.25 per nucleotide). The motif score of a chip-seq peak is the maximum LLR of any window in that peak.

### 2.5 Target gene identification and GO enrichment analysis

We identify putative target genes of each TF as genes located either within 2 kbp sequentially of a TFBS for that factor, or on a spatially proximal region to that TFBS. Within each group, we consider the functions of target genes, using Gene Ontology (GO) functional enrichment analysis and disease trait enrichment analysis.

Based on the distributions of putative TF regulators per gene (supplementary Fig. A.22), genes that are targets for at least five TFs from one group, and at most three TFs from the other group, are taken to be target genes for the former group. We perform GO term enrichment analysis for biological processes, and molecular functions using DAVID [24] with background of all human genes. To reduce the multiple testing burden, we use GO-FAT terms to filter broad GO terms.

### 2.6 Datasets used

The chromatin interaction datasets and uniformly processed ChIP-seq peaks generated by ENCODE project used in the study are given in the supplementary Tables A.1 and A.2.

## 3 Results

### 3.1 TFs fall in two broad groups

**Figure 2:**
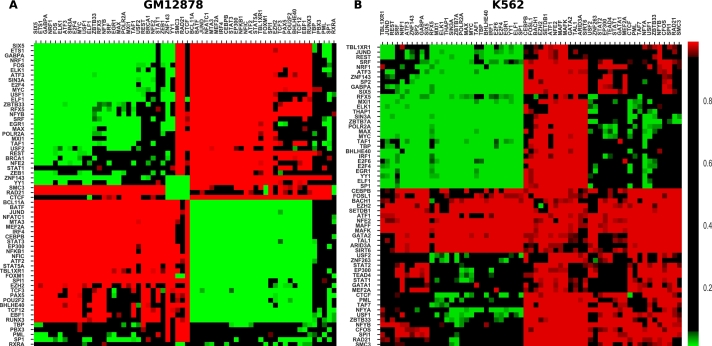
Clustered *q*-value heatmap showing attracting and repelling TF pairs in green and red respectively as described in Methods for (A) GM12878 and (B) K562 cell lines using ChIA-Pet Pol-II data.

We explored co-binding of TFs in spatially proximal chromatin regions identified from ChIA-PET Pol-II of GM12878 and K562 for 62 and 61 TFs respectively using their TFBS data from ChIP-seq peaks as described in Methods (Fig. 2).

In the GM12878 cell line, TFs segregate into two groups (Fig. 2A). TFs in each group tend to attract other members of the same group (green, top left and bottom right of heatmap) but repel members of the other group (red, top-right, bottom-left). We call the top-right group “Group 1” and the bottom-right group “Group 2”.

A similar study, using a different methodology and a different (Hi-C) dataset, was performed by Ma et al. (2018) [15]. Supplementary Fig. A.3 compares the results of the two studies; with a very few exceptions, our results are consistent with those of Ma et al. For completeness, we also provide the results of our method on the same Hi-C dataset used by Ma et al. (Fig. A.4). Again, the results are consistent on the subset of TFs that they study (Fig. A.5); however, on the larger set of TFs that we use, there are some notable differences and the biclustering into two groups is not as clear, and certain associations that are experimentally known (e.g., association of CTCF with cohesin subunits RAD21 and SMC3) are recovered in the ChIA-PET data but not in the Hi-C data. As noted in Introduction, we focus on ChIA-PET data partly because TF binding to ChIA-PET regions is more likely to be functionally relevant.

Xie et al. [25] studied co-localization of 50 TFs. A simple hierarchical clustering of these colocalization scores reveals a structure in good agreement with the two TF groups that we see, and boxplots of the co-localization scores show significantly lower scores for inter-group TF pairs compared to intra-group pairs (supplementary Figs. A.6 and A.7).

In the K562 cell line, we do not observe two distinct classes of attracting TFs as in GM12878; instead, one attracting group emerges on the top-left, and most other TF pairs repel. However, as discussed further below, many of the characteristics of Group 1 from GM12878 are retained in the one attracting group in K562. We further discuss the differences between Group 1 and Group 2 in later subsections.

**Figure 3:**
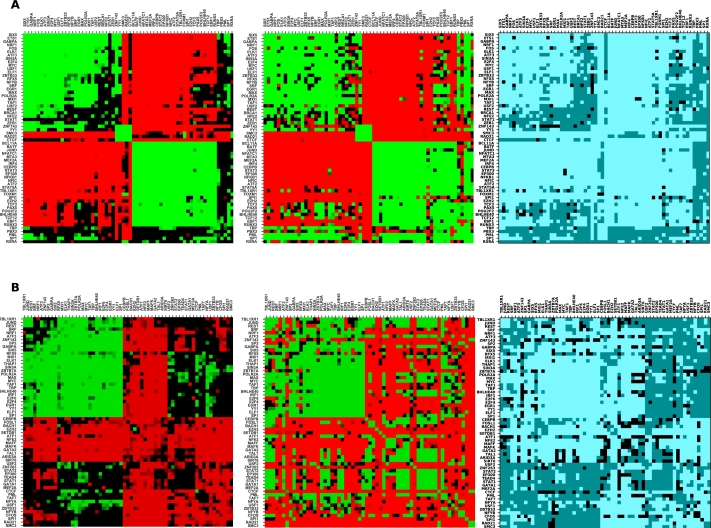
Top (A) and bottom (B) panels show the co-occurrence pattern in spatially proximal and sequentially contiguous regions for GM12878 and K562 cell lines respectively. The left heatmap in both panels corresponds to spatial co-occurrence, the middle to sequential co-occurrence, while the right heatmap is a comparison of these, coded as follows: both significant, in agreement: bright blue; both significant, in disagreement: black; one or both insignificant: dark blue.

Notably, in both GM12878 and K562, CTCF and cohesin subunits SMC3 and RAD21 almost universally repel other TFs. In GM12878 ChIA-PET and (below) in sequentially contiguous binding in multiple cell lines, these proteins co-occur among themselves, but we do not observe this in K562 or in GM12878 Hi-C data. Cohesin and CTCF have previously been reported to co-occur in spatially proximal regions and this co-occurrence is believed to be a critical for the formation of 3D chromatin loops [26], [27]. Pairs of CTCF motifs are found in a divergent orientation near cohesin, and the chromatin “loop extrusion model” has been proposed for chromatin organization via “topologically associated domains” (TADs). In this model, a loop of DNA is pushed through a cohesin ring, until it is hindered by CTCF molecules bound at the motif sites. This has been recently observed *in vitro* [28]. Our observations in GM12878 ChIA-PET data support the proposed interplay of cohesin and CTCF, and further suggest that the binding regions of cohesin in particular tend not to be in close spatial proximity with either promoters or other distal regulatory regions where other TFs tend to bind. Fig. 3, in the next section, suggests that in addition to not being in spatial proximity of other TFBS, cohesin subunits are also not in close sequential proximity of other TFBS, but consistently co-occur with CTCF and each other. We speculate that the lack of consistent co-occurrence in Hi-C data may be due to the multiple roles and genome-wide binding exhibited by CTCF.

In addition to TFs, we considered histone marks in our GM12878 analysis. The resulting attracting and repelling marks and TFs are shown in supplementary Fig. A.8. We observe that histone marks associated with active promoters or enhancers i.e. H3K4me1, H3K4me2, H3K4me3, H3K27ac, and H3K9ac significantly co-occur among themselves even in spatially proximal regions similar to what has been reported in various previous studies based on sequentially contiguous regions of the genome [29], [30], [31], [32] and also co-occur with factors TBP, PML, and SP1 which are usually associated with the promoter regions. Also, these histone marks significantly repel their antagonistic marks H3K27me3 and H3K9me3.

Previous chromatin interaction studies have shown that the genome is primarily partitioned into distinct two compartments A and B, characterised by open and closed chromatin respectively [4], [27]. It would be interesting to know if these compartments have any relation, or more importantly, if they contribute to the segregation of TFs that we observed in our investigation. To this end, we investigated the co-occurrence of TFs in the A and B compartments separately by classifying the spatial chromatin interactions falling into these compartments. But, we do not observe any such correlation of genome compartments with clusters of TFs in terms of their co-occurrence. The TF pair co-occurrence behaviour is given in supplementary Fig. A.9A and A.9B for A and B compartments respectively. The co-occurrence behaviour is similar in both compartments, but the numbers of significant attracting or avoiding TF pairs are fewer in the B compartment, as expected, it being generally inactive with a closed chromatin structure.

#### 3.1.1 Sequential co-occurrence patterns largely mirror spatial co-occurrence

Prior to the advent of high-throughput chromatin interaction experiments such as Hi-C, co-occurrence of transcription factors was studied in sequentially contiguous regions such as promoters and enhancers [33], [34], and we asked whether the same patterns of attraction or repulsion occur within contiguous regulatory sequence. We repeated the analysis using the same regions from the ChIA-PET data, and the same randomization method, but this time considering co-occurrence within rather than across interacting regions. It turns out that most TF pairs exhibit the same qualitative behaviour as in spatially proximal regions; however, a few TF pairs show different qualitative behaviour (Fig. 3). These include interactions across groups: in particular, factors of Group 1 (SRF, EGR1, MAX, MXI1, TAF1) attract factors of Group 2 (TCF3, PAX5, POU2F2, BHLHE40, TCF12), visible as a green block in Fig. 3A (middle). Their pairwise spatial interactions are insignificant but they show significant co-occurrence in sequentially contiguous regions.

In GM12878, factors such as TBP, PML, SP1 which are usually enriched at the promoter regions attract almost all other TFs used in the study sequentially, but do not show any significant behaviour in spatially proximal regions, highlighting the promoter specific binding of these factors. In K562 cell line, as mentioned in the previous section, we observed only one main cluster of TFs attracting each other, but the other cluster of TFs, which do not attract spatially, do in many cases attract in sequentially contiguous regions. Overall, the analysis in spatial and sequential regions and comparison between them substantiate the evidence of two clusters of TFs present and suggest their potential role in the regulatory aspects.

#### 3.1.2 Motif instances attract and repel similarly to TFBS

Our results above from the TF ChIP-seq binding data show considerable similarities and differences across cell lines in pattern of significant co-occurrence or avoidance in both spatially proximal and sequential contiguous regions. TFs in general bind to the genomic regions in a sequence specific manner and these short specific sequences are represented as sequence motifs. Motif instances of a given TF are present at several locations of genome, but only at few of those sites does the TF bind in a given cell type depending on additional factors. We have so far considered binding events identified via ChIP-seq experiments. Here we ask whether a similar co-occurrence pattern occurs at a motif level, even though motif instances are generally poor indicators of tissue-specific TF binding.

We repeat the same analysis here, using motif occurrence rather than ChIP-seq binding information. Motif instances are predicted with FIMO [20] using motifs for TFs from the JASPAR database [21] as bundled with the MEME suite [22].

**Figure 4:**
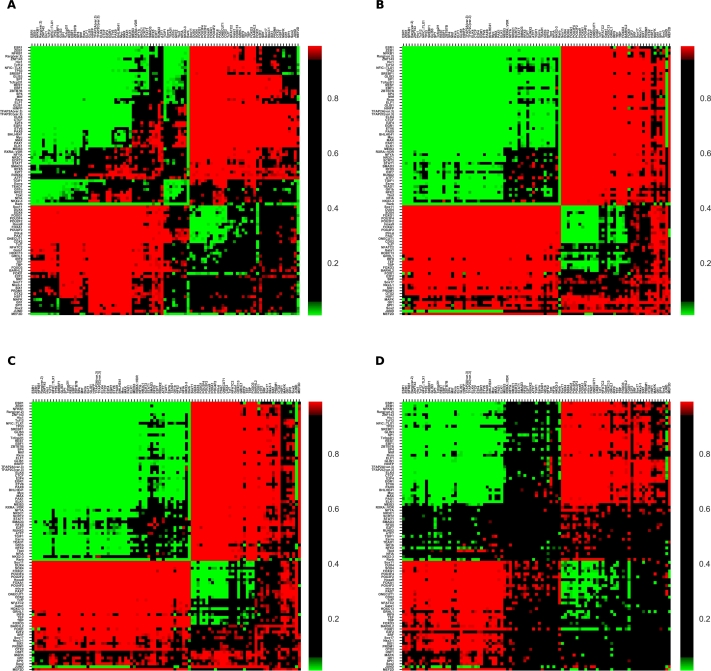
Comparison of motif co-occurrence in four cell lines: (A) GM12878, (B) K562, (C) MCF7 and (D) HeLa-S3. For ease of comparison, the order of factors from the clustering in GM12878 is used in all four subfigures.

Motif information is available for a large number of TFs beyond the ones for which ChIP-seq data is available. We can study motif co-occurrence in various cell lines; while motif matches (unlike chip-seq peaks) are independent of cell lines, the chromatin contact information is cell line-dependent. We cluster JASPAR TF motifs into 93 groups, and pick a representative PWM for each group for site prediction (Methods). Using these 93 distinct motifs, we observe strongly similar patterns of co-occurrence of motif sites in spatially proximal regions for four cell lines: GM12878(A), K562(B), MCF-7(C) and HeLa-S3(D), as shown in Fig. 4.

We perform a similar analysis with 286 clustered motif models from Vierstra et al. (2020) [35], which is shown in supplementary Fig. A.12. We again selected the most informative PWM for each motif cluster, to avoid weak low-confidence predictions. The clustering of attracting and repelling TFs is visually similar.

Motif co-occurrence in sequentially contiguous regions too is broadly similar to the spatially proximal regions, in all the four cell lines examined. This can be seen in the supplementary Figs. A.13, A.14, A.15, and A.16 for GM12878, K562, HeLa-S3, and MCF7 cell lines respectively. However, there are many more significant examples both of attraction and of repulsion in sequentially contiguous regions, suggesting perhaps a greater degree of combinatorial control within promoters and enhancers.

### 3.2 The two main TF groups interact differently with proteins, DNA, and genes

In GM12878 TFs separate into two groups that attract within a group, but repel across groups, with some exceptions. In K562 there is one group that mutually attracts (and largely mirrors Group 1 in GM12878), and another that repels most other TFs in our dataset. The two groups in each case exhibit differences in their interactions with other proteins, DNA binding locations, and downstream gene targets.

#### 3.2.1 PPI interactions are enriched in intra-group TF pairs in GM12878

We examined physical interactions of TF-TF pairs using the Human Integrated Protein-Protein Interaction Reference (v2.2) database (HIPPIE) [36]. We observe enrichment of physical interaction among attracting TF pairs (92 out of 539 i.e. ∼0.17) compared to avoiding TF pairs (40 out of 523 i.e. ∼0.07) indicating that attracting TF pairs are significantly more likely to interact physically than avoiding pairs ($p=1.98\times 10^{-6}$, hypergeometric test). There is no significant difference between Group 1 TF pairs and Group 2 TF pairs (interacting fraction ∼0.15 and ∼0.18 respectively).

#### 3.2.2 Domain-domain interactions are enriched in attracting TF pairs

To further examine the possibility of undocumented protein-protein interactions, we considered the domain structures of the TFs and looked for possible domain-domain interactions among all TF pairs present in the study, using the database of three-dimensional interacting domains (3did) [37]. Any pair of TFs containing respective interacting domains, was considered “potentially interacting”. Attracting TF pairs in both cell lines show significant enrichment over avoiding TF pairs for potential physical interaction (Table 1, Table 2).

**Table 1:** The number of possible physical interactions from the literature of domain-domain physical interactions among attracting and avoiding TF pairs for GM12878 cell line. Attracting pairs are significantly more likely to have possible domain-domain interactions (*p* = 2.17 × 10^−9^, hypergeometric test).

	Attracting TF pairs	Avoiding TF pairs	Total
potential physical interactions	131	61	192
No potential physical interactions	598	718	1316
Total	729	779	1508

**Table 2:** Similar to Table 1, for K562 cell line. Attracting pairs are significantly more likely to have possible domain-domain interactions (*p* = 7.65 × 10^−12^, hypergeometric test).

	Attracting TF pairs	Avoiding TF pairs	Total
potential physical interactions	79	24	103
No potential physical interactions	369	517	886
Total	448	541	989

#### 3.2.3 Internal nodes in PPI pathways largely differ in Group 1 and Group 2 for GM12878

**Figure 5:**
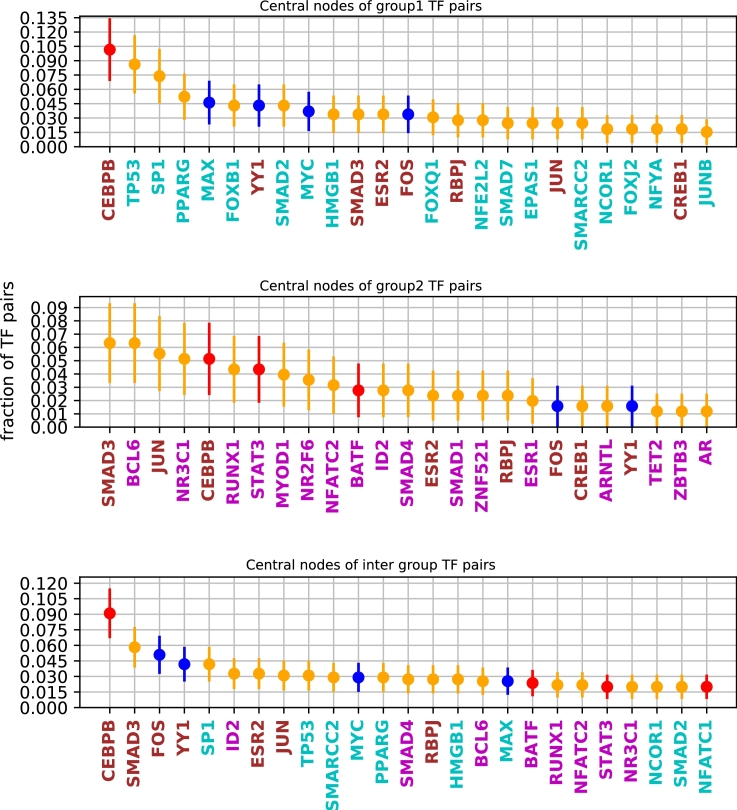
The TFs most frequently occurring as internal nodes in the shortest PPI pathway between TF pairs, for pairs from Group 1 (top), Group 2 (middle), and cross-group(bottom panel). The blue, red markers are Group 1, Group 2 TFs respectively and yellow are TFs not present in our co-occurrence data. TFs in cyan text occur as central nodes within Group 1 only, in magenta are central nodes within Group 2 only, and brown occur as central nodes in both groups.

We next looked at TFs that may be binding indirectly, via co-factors, or may have shared functionality because of presence on a common pathway. We considered the shortest PPI pathway for each pair of TFs. Specifically, we asked what TFs (not necessarily in our list of 62) occur in the shortest interaction pathway between that pair of TFs, and evaluated the frequency of occurrence of each such “internal node”. We only considered annotated TFs, and not other proteins (Fig. 5). Interestingly, the frequent internal nodes are largely unique for each group. Moreover, the few TFs common to these groups tend to occur as internal nodes of internal-group TF pairs, suggesting possible roles as co-activators or co-repressors. Taken together, this analysis suggests that the two groups have distinct structural underpinnings.

While pairwise PP interactions are mechanistic, higher-order interactions, along such paths, need not indicate a physical interaction, but may be indicative of a functional association. However, given that many of these proteins are widely expressed (e.g., housekeeping proteins), physical interactions are possible too. The observed differences in frequent nodes in the shortest PPI path are indicative of a functional difference but await detailed investigation.

It is worth noting that the internal nodes we consider here need not be TFs. We examined a recent study by Göös et al. (2022) [38] on human TF interaction networks, but this TF-specific data was too sparse for this sort of analysis.

#### 3.2.4 Group 1 TFs bind closer to promoters than Group 2 TFs

**Figure 6:**
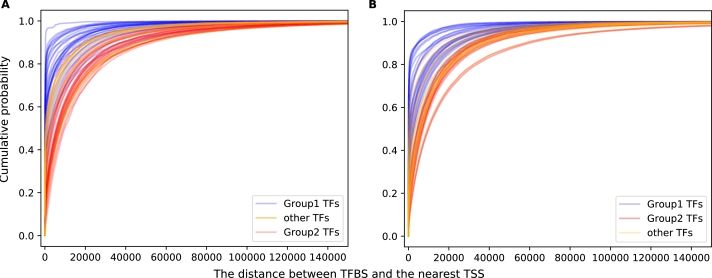
(A) The distance between TFBS and the nearest TSS, and the cumulative probability for sites to occur within that distance, for Group 1 TFs (blue), Group 2 TFs (red) and other TFs (yellow), in (A) GM12878 and (B) K562.

Fig. 6 plots the cumulative distribution of ChIP-seq peak distance from the nearest transcription start site (TSS) for Group 1, Group 2, and ungrouped TFs, in (A) GM12878 and (b) K562. Group 1 TFs tend to bind closer to the TSS than Group 2 or ungrouped TFs. This is consistent with what Ma et al. (2018) [15] report.

In supplementary Fig. A.19 we show that the two groups of TFs also differ in the GC content of their binding peaks. Previously it has been reported that enhancers tend to interact with promoters of similar GC composition [39], [40]. Our results are consistent with this and suggest that, in general, TFs that attract in 3D chromatin tend to bind to regions with similar GC content.

#### 3.2.5 Group 1 target genes are enriched for housekeeping functions, Group 2 for tissue-specific functions

We identify putative target genes for each TF, and perform GO analysis, as described in Methods.

**Figure 7:**
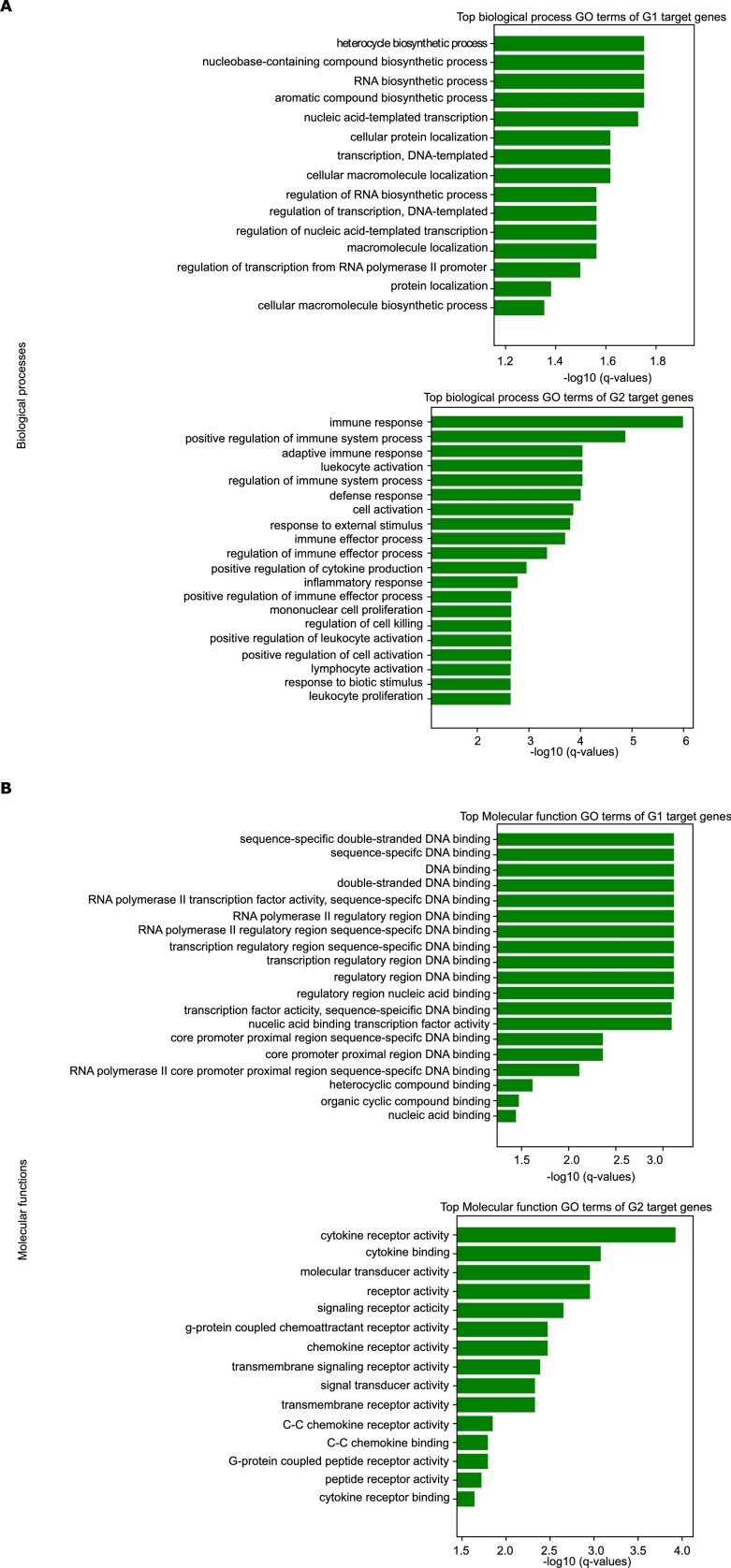
(A) shows the enriched biological process GO terms for the Group 1 and Group 2 target genes. (B) Similarly, enriched GO molecular function terms are shown for Group 1 and Group 2 target genes.

We find that Group 1 target genes are enriched for housekeeping functions such as biosynthetic processes, transcription, and protein and macromolecule localization, while Group 2 target genes are enriched for immune/inflammatory response (Fig. 7A).

We specifically assessed whether Group 1 target genes are enriched for housekeeping genes using a database of known housekeeping genes [41]. Group 1 target genes are significantly enriched over Group 2 target genes for housekeeping genes (hypergeometric test p-value $3.7\times 10^{-16}$). The enrichment is not highly sensitive to the target gene selection criteria (for example, requiring 3 TFs from one group and 0 from the other yields similar results).

The biological process enrichment is corroborated by molecular function enrichment. The molecular functions of Group 1 target gene involve mostly DNA binding events, while Group 2 genes are enriched for receptor activity of cytokines and chemokines involved in immune responses (Fig. 7B).

Next, we assessed the group-specific target genes for association with disease traits from the GWAS catalog [42], only considering the traits associated with at least 5 genes. The target genes of each group were examined for significant overlap with these traits (Fisher exact test p<0.05). Consistently, Group 2 targets were enriched for inflammatory disease, and immunological diseases involving lymphocytes, leukocytes, and neutrophils, while Group 1 genes are not particularly enriched for any particular diseases (supplementary Fig. A.24). Enrichment of immune response and traits in Group 2 targets is especially interesting considering that GM12878 is a lymphocytic cell line.

We performed a similar analysis for K562 cell line treating the large group of mutually attracting TFs as Group 1 and the remainder as Group 2. Again, Group 1 target genes are enriched for housekeeping functions such as transcription, metabolism, and transport (hypergeometric test p-value $4.5\times 10^{-5}$). Group 2 target genes are not particularly enriched for pathways or functions (supplementary Fig. A.25).

Finally, in the HeLa-S3 cell line we see a similar segregation into two groups as in GM12878, but Group 1 is much larger than Group 2 (supplementary Fig. A.10). Again, Group 1 is significantly enriched for housekeeping functions ($p=4.9\times 10^{-17}$) (supplementary Fig. A.26). Group 2 is enriched for protein ubiquitination and various metabolic processes, but with little overlap to Group 2 of GM12878.

Both the similarity and differences across the cell lines in terms of TF groupings and their target functions are worth noting. GM12878 is a lymphoblastoid cell line derived from the blood of a healthy female donor, K562 is lymphoblasts isolated from the bone marrow of a chronic myelogenous leukemia patient, and HeLa-S3 is a cervix carcinoma cell line. While Group 1 TF targets in all three cell lines are enriched for housekeeping functions, Group 2 of TFs is most prominent in GM12878, derived from a healthy donor, and their targets are enriched for immune function. In contrast, leukemia-derived K562 lacks a functionally coherent Group 2 TFs, which may suggest a loss of lineage-specification in leukemic lymphoblasts. HeLa-S3 has a smaller Group 2 than GM12878 with a divergent set of, potentially lineage-specific, target functions.

### 3.3 Motif strength correlates with spatial interaction

Previous studies have demonstrated the significance of long-range spatially proximal regions in aiding *in vivo* TF binding at weaker motif sites by containing several homotypic motif sites in spatially clustered genomic regions [14], [15]. We extend the investigation to spatial interactions with other genomic regions, regardless of whether there are TFs binding to those other regions.

For each TF we consider the most informative PWM available in JASPAR [21] (Methods). Individual binding sites may have stronger or weaker matches to the PWM, which is an estimate of their binding affinity to the TF. We use the log likelihood ratio (Methods) as the “motif score” for a site, a measure of the likely “strength” of binding at that site. For each TF, we consider peaks containing sites whose motif scores are among the top 10% as “peaks with strong motifs”. We then compare the fraction of peaks with strong motifs between two groups of peaks—those that are isolated (occurring in regions without any spatial chromatin interactions) and those in spatially interacting regions.

**Figure 8:**
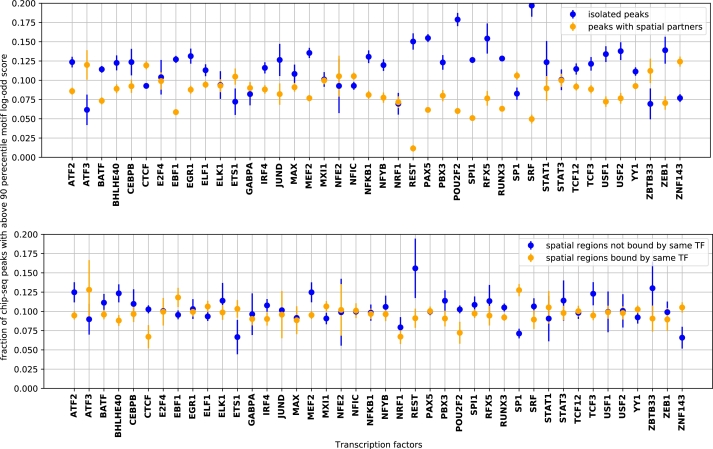
Spatially isolated ChIP-seq peaks mostly have a higher fraction of strong motifs than peaks within spatial clusters (top panel). For peaks within spatial clusters, when the same TF binds to a spatially-interacting region, the associated motif is weaker in 24 out of 42 cases (bottom panel).

We observe for almost all factors, spatially isolated TFBS exhibit a significantly greater fraction of strong motifs (Fig. 8A). If we consider whether the spatially interacting region is bound by the same TF or is not (but possibly bound by another TF), in the majority of cases binding by the same TF is associated with a weaker motif, but this is highly variable (Fig. 8A). A similar trend is observed in K562 cell line (supplementary Fig. A.20). This suggests that spatial interactions enable binding by TFs to weaker motifs, even in the absence of homotypic binding or the presence of heterotypic binding as was suggested previously.

Of note, the ChIP-seq peak scores do not show a similar correlation with isolated/interacting regions (supplementary Fig. A.21). This enforces the point that the presence of 3D-proximal regions enables TF binding *despite* a weak motif. A comparable observation for homotypic TFs was made in Malin et al. (2015) [14].

#### 3.3.1 A consensus TF-TF co-occurrence network

**Figure 9:**
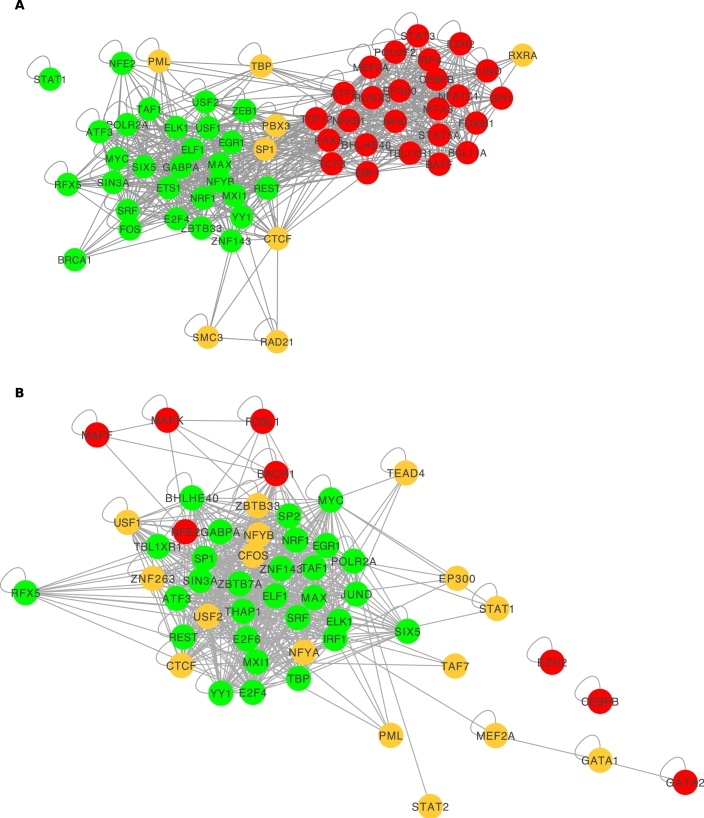
High confidence consensus networks built using the TF pair co-occurrence observations from all four methods (spatial or sequential, binding or motif instance) for (A) GM12878 and (B) K562 cell lines.

Given the variability across different datasets and approaches, we focus here on widely conserved interactions. Using the co-occurrence patterns for binding events and motifs that we see in spatially and sequentially contiguous regions, we propose a consensus network for TF pair co-occurrence. We use both motif and experimental (ChIP-seq) binding events, and both spatially proximal and sequentially contiguous regions, giving us four datasets; and we require that TF pairs attract in at least two of these four datasets. The consensus network is shown in Fig. 9A and 9B for GM12878 and K562 cell lines respectively. A comparison of observations from all four types is shown in supplementary Figs. A.17 and A.18. This high confidence consensus network further highlights the segregation of TFs into two groups with certain TFs such as TBP, PBX3, SP1, REST, TCF12, TCF3, and PAX5, acting as intermediates, while cohesin subunits SMC3 and RAD21 are outliers.

## 4 Discussion

We present a methodology to assess significant co-occurrence, and significant avoidance, between pairs of transcription factors in both linear as well as spatially proximal regions in genome. We find that most TF pairs attract or repel (FDR q≤0.05) in every cell type that we examined. The cohesin subunits RAD21 and SMC3 tend to repel most other factors across cell lines. CTCF tends to repel many factors, but attracts others. This suggests that regions where cohesin rings form are not transcriptionally active [43], [44], [45], [46], [47], while CTCF, which plays multiple roles [48] including chromatin organization, insulator function, and transcriptional regulation, can attract or avoid other factors based on function. A few factors, notably promoter-associated factors such as SP1 and TBP, co-occur widely with factors in both groups.

While our method and our dataset (ChIA-PET) differs from Ma et al. (2018) [15] (who used Hi-C data), our predictions largely agree with theirs in GM12878 cells for the TFs that they consider. For completeness, we also tested on the same Hi-C dataset as they did, with similar results (supplementary Fig. A.5). We focus on Pol-II ChIA-PET rather than Hi-C for two main reasons: it is higher-resolution (≈ 1 kb, compared to 5 kb for Hi-C); and TF binding to Pol-II ChIA-PET regions is more likely to be functional (since these regions are more likely to be functional promoters or enhancers).

Our method considers the network connectivity properties when generating random networks, and involves fewer parameter choices. We consider many more TFs, across multiple cell lines and multiple data sets. We additionally include histone marks associated with various chromatin states in this analysis. We perform and compare with similar analyses for sequentially-adjacent binding and for co-occurrence of motifs in spatially adjacent sequence.

TF co-occurrence pattern is corroborated by co-occurrence of histone marks in GM12878: marks associated with active promoters/enhancers co-occur with one another and also with TBP, PML and SP1 and a large group of TFs, while repelling a smaller group of other histone marks, including marks associated with heterochromatin. Heterochromatin marks repel other histone marks and most TFs. In GM12878, we also find substantial agreement with a previous analysis based on a different methodology [15].

Sequentially proximal binding shows largely the same pattern of co-occurrence as the spatially proximal binding but with greater significance. Furthermore, motif instances too show co-occurrence similar to ChIP-seq binding events, overall suggesting a potentially coordinated evolution of cis elements with the chromatin structure. We synthesise the various co-occurrence data (spatial and sequential; TF binding and motif instance) into a consensus co-occurrence network that illustrates these points (Fig. 9A).

We find that the attraction-repulsion patterns and the separation into groups is reflected in various biological properties of the TF pairs and groups, including proximity from TSS, protein interactions networks, domain-domain interactions, and function of downstream target genes. These too exhibit similar trends across cell lines, with some differences. The similarities suggest that basic transcriptional machinery depends on the chromatin structure bringing appropriate regions together; while the differences that do occur suggest that chromatin state plays an important function in cell-type differentiation. Specific differences in the revealed TF groups among K562, HeLa-S3 and GM12828 suggest that while Group 1 TFs associated with housekeeping functions are conserved between the three cell lines, the Group 2 TFs associated with lineage-specific functions are disrupted specifically in the malignant lymphoblast-derived K562 and cervical carcinoma-derived HeLa-S3.

Finally our method provides a platform for testing heterotypic as well as homotypic spatial interactions of TFs. While previous studies have suggested the role of homotypic binding in spatially clustered regions in boosting *in vivo* binding [14], our analysis shows that proximity to other regulatory regions is associated with stronger *in vivo* binding, regardless of TF binding in the spatially proximal regions.

Overall, our work indicates a complex interplay of TF binding and chromatin structure, some of which we elucidate in detail, while much remains to be discovered in future. Our tool, ChromTogether, will facilitate future studies in this field.

## CRediT authorship contribution statement

Rakesh Netha Vadnala: Performed the experiments; Analyzed and interpreted the data; Contributed reagents, materials, analysis tools or data; Wrote the paper. Sridhar Hannenhalli, Leelavati Narlikar, Rahul Siddharthan: Conceived and designed the experiments; Wrote the paper.

## Declaration of Competing Interest

The authors declare that they have no known competing financial interests or personal relationships that could have appeared to influence the work reported in this paper.

## Data Availability

Data included in article/supp. material/referenced in the article.

## Appendix A Supplementary material

MMC Supplementary Information. MMC
