# Supplementary material for: Transcription factors organize into functional groups on the linear genome and in 3D chromatin
Source: Heliyon. 2023 Jul 17;9(8):e18211. doi: 10.1016/j.heliyon.2023.e18211 (PMC10382302; doi:10.1016/j.heliyon.2023.e18211)
Supplement: MMC — Supplementary Information. [file mmc1.pdf]

Appendix A. Supplementary Information

Materials & Methods

Cell line	Data Set	Accession Number
GM12878	ChIA-PET (RNAPII)	GSM1872887
K562	ChIA-PET (RNAPII)	GSM970213
MCF7	ChIA-PET (RNAPII)	GSM970209
HeLa-S3	ChIA-PET (RNAPII)	GSM1872889

Table A.1: Accession IDs of chromatin interaction data sets used in the study

**GM12878**	
ATF2	EncodeEH002306
ATF3	EncodeEH001562
BATF	EncodeEH001479
BCL11A	EncodeEH001486
BHLHE40	EncodeEH002025
BRCA1	EncodeEH001830
CEBPB	EncodeEH003212
CTCF	EncodeEH000029
CTCF	EncodeEH000394
CTCF	EncodeEH000532
CTCF	EncodeEH001851
E2F4	EncodeEH002867
EBF1	EncodeEH001832
EGR1	EncodeEH002328
ELF1	EncodeEH001617
ELK1	EncodeEH002851
EP300	EncodeEH002037
EP300	EncodeEH002824
ETS1	EncodeEH001564
EZH2	EncodeEH002411
FOS	EncodeEH000622
FOXM1	EncodeEH002529
GABPA	EncodeEH001462
IRF4	EncodeEH001484
JUND	EncodeEH000639
MAX	EncodeEH002806
MAZ	EncodeEH002852
MEF2A	EncodeEH001565
MTA3	EncodeEH002329
MXI1	EncodeEH002026
MYC	EncodeEH000547
NFATC1	EncodeEH002307
NFE2	EncodeEH001808
NFIC	EncodeEH002343
NFYB	EncodeEH002065
NRF1	EncodeEH001846
PAX5	EncodeEH001489
PAX5	EncodeEH001495

PBX3	EncodeEH001477
PML	EncodeEH002308
POLR2A	EncodeEH000626
POLR2A	EncodeEH001517
POU2F2	EncodeEH001475
RAD21	EncodeEH000749
REST	EncodeEH002314
RFX5	EncodeEH001810
RUNX3	EncodeEH002330
RXRA	EncodeEH001541
SIN3A	EncodeEH002868
SIX5	EncodeEH001542
SMC3	EncodeEH001833
SP1	EncodeEH001496
SPI1	EncodeEH001476
SRF	EncodeEH001464
STAT1	EncodeEH001852
STAT3	EncodeEH001811
STAT5A	EncodeEH002321
TAF1	EncodeEH001478
TBL1XR1	EncodeEH002853
TBP	EncodeEH001798
TCF12	EncodeEH001485
TCF3	EncodeEH002315
USF1	EncodeEH001468
USF2	EncodeEH001812
WRNIP1	EncodeEH001787
YY1	EncodeEH000695
YY1	EncodeEH001657
ZBTB33	EncodeEH001488
ZEB1	EncodeEH001645
ZNF143	EncodeEH001853
**K562**	
ARID3A	EncodeEH002861
ATF1	EncodeEH002865
ATF3	EncodeEH000700
BACH1	EncodeEH002846
BHLHE40	EncodeEH001857
CEBPB	EncodeEH001821
CEBPB	EncodeEH002346
CTCF	EncodeEH000042
CTCF	EncodeEH000399
CTCF	EncodeEH000535
CTCF	EncodeEH002279
CTCF	EncodeEH002797
E2F4	EncodeEH000671
E2F6	EncodeEH000676
E2F6	EncodeEH001598
EGR1	EncodeEH001646
ELF1	EncodeEH001619
ELK1	EncodeEH003356

EP300	EncodeEH002086
EP300	EncodeEH002834
ETS1	EncodeEH001580
EZH2	EncodeEH002089
FOS	EncodeEH000619
FOS	EncodeEH001207
FOSL1	EncodeEH001637
GABPA	EncodeEH001604
GATA1	EncodeEH000638
GATA2	EncodeEH000683
GATA2	EncodeEH001208
GATA2	EncodeEH001576
IRF1	EncodeEH001865
IRF1	EncodeEH001866
IRF1	EncodeEH002798
IRF1	EncodeEH002799
JUND	EncodeEH001211
JUND	EncodeEH002164
MAFF	EncodeEH002804
MAFK	EncodeEH001844
MAX	EncodeEH001605
MAX	EncodeEH002869
MEF2A	EncodeEH001663
MXI1	EncodeEH001827
MYC	EncodeEH001867
MYC	EncodeEH002800
NFE2	EncodeEH000624
NFYA	EncodeEH002021
NFYB	EncodeEH002024
NRF1	EncodeEH001796
PML	EncodeEH002320
POLR2A	EncodeEH000704
POLR2A	EncodeEH000727
RAD21	EncodeEH000649
RAD21	EncodeEH001585
REST	EncodeEH001638
RFX5	EncodeEH002033
SETDB1	EncodeEH000677
SIN3AK20	EncodeEH001607
SIRT6	EncodeEH000681
SIX5	EncodeEH001483
SMC3	EncodeEH001845
SP1	EncodeEH001578
SP2	EncodeEH001653
SPI1	EncodeEH001482
SRF	EncodeEH001600
STAT1	EncodeEH000760
STAT1	EncodeEH000761
STAT2	EncodeEH000665
STAT5A	EncodeEH002347
TAF1	EncodeEH001582
TAF7	EncodeEH001654

TAL1	EncodeEH001824
TBL1XR1	EncodeEH002848
TBL1XR1	EncodeEH002849
TBP	EncodeEH001825
TEAD4	EncodeEH002333
THAP1	EncodeEH001655
USF1	EncodeEH001583
USF2	EncodeEH001797
YY1	EncodeEH000684
YY1	EncodeEH001584
YY1	EncodeEH001623
ZBTB33	EncodeEH001569
ZBTB7A	EncodeEH001620
ZNF143	EncodeEH002030
ZNF263	EncodeEH000630
**HeLa-S3**	
BRCA1	EncodeEH001814
BRF1	EncodeEH000764
BRF2	EncodeEH000765
CEBPB	EncodeEH001815
CHD2	EncodeEH002027
CTCF	EncodeEH000398
CTCF	EncodeEH000541
CTCF	EncodeEH001012
E2F1	EncodeEH000688
E2F1	EncodeEH000699
E2F4	EncodeEH000689
E2F6	EncodeEH000692
ELK1	EncodeEH002864
ELK4	EncodeEH001753
EP300	EncodeEH001820
EZH2	EncodeEH003086
FAM48A	EncodeEH001855
FOS	EncodeEH000647
GABPA	EncodeEH001504
GTF2F1	EncodeEH001816
GTF3C2	EncodeEH000747
IRF3	EncodeEH001788
JUN	EncodeEH000746
JUND	EncodeEH000745
MAFK	EncodeEH002856
MAX	EncodeEH002830
MAZ	EncodeEH002855
MXI1	EncodeEH001826
MYC	EncodeEH000542
MYC	EncodeEH000648
NFYA	EncodeEH002066
NFYB	EncodeEH002067
NR2C2	EncodeEH000687
NRF1	EncodeEH000723
POLR2A	EncodeEH000597

POLR2A	EncodeEH000613
POLR2A	EncodeEH001021
POLR2A	EncodeEH001474
POLR2A	EncodeEH001838
PRDM1	EncodeEH001817
RAD21	EncodeEH001789
RCOR1	EncodeEH002844
REST	EncodeEH001629
RFX5	EncodeEH001818
RPC155	EncodeEH000766
SMC3	EncodeEH001839
STAT1	EncodeEH000614
STAT3	EncodeEH001799
TAF1	EncodeEH001505
TBP	EncodeEH001790
TCF7L2	EncodeEH002069
TCF7L2	EncodeEH002813
USF2	EncodeEH001819
ZKSCAN1	EncodeEH002857
ZNF143	EncodeEH002028
ZNF274	EncodeEH001763

Table A.2: Accession IDs of the uniformly processed chip-seq peaks generated by ENCODE project used in the study

The commands for various external tools that were used in the study are given below.

For scanning presence of motif instances in chromatin regions, FIMO [20] was used with following command:

```
fimo -o <output-directory> <TF-motif-meme-file> <fasta-sequence-file-of-chromatin-regions>
```

For finding the similarity between all the nonredundant JASPAR motifs, we used the TOMTOM [23] external tool:

```
tomtom <query-motif-file> <target-motif-file>
```

Network statistics

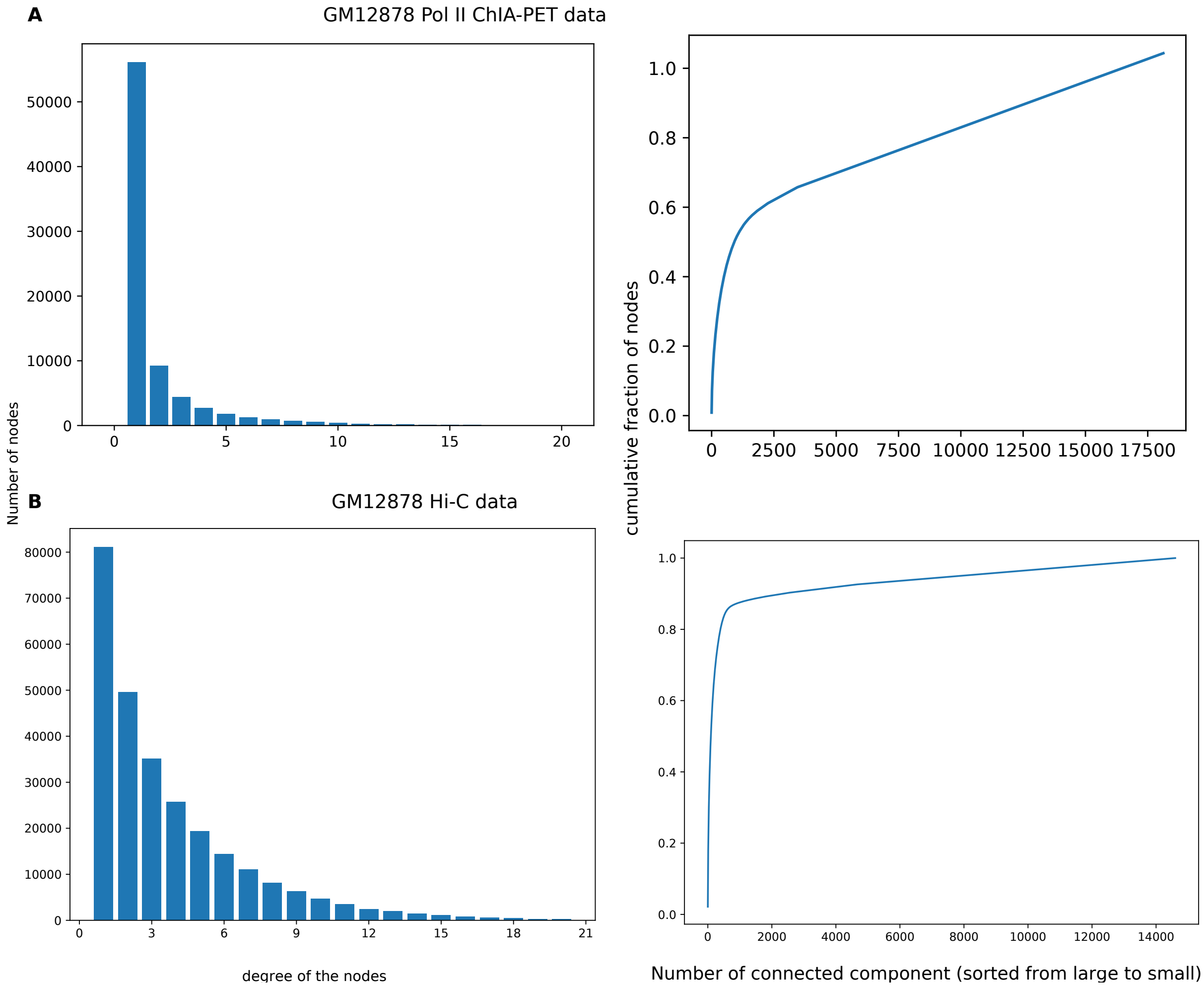

Figure A.1: The figure shows the distribution of degree of nodes (left) and cumulative size of isolated components sorted from larger to smaller component (right) of the interaction network constructed using Pol II ChIA-PET data in (A) and using Hi-C data in (B). Most nodes have degree 5 or less; therefore we choose to conserve the degree of such nodes in randomization. For degree 6-10 we conserve the degree within ± 2, and the nodes with degree > 10 are considered equivalent for randomization purposes.

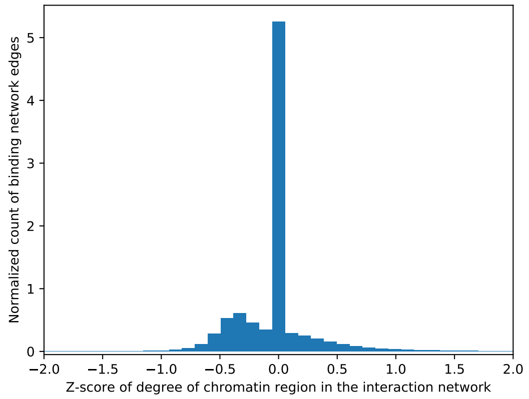

Figure A.2: The distribution of z-scores of degree of regions with chip-seq peaks during our randomization process. For example, for each chip-seq peak the deviation of degree of region in the interaction network to which it is originally bound and degree of region to which it is assigned throughout 1000 randomization steps is calculated. The randomization process fairly conserves the degree of nodes.

Comparison with Ma et al. and Xie et al. study

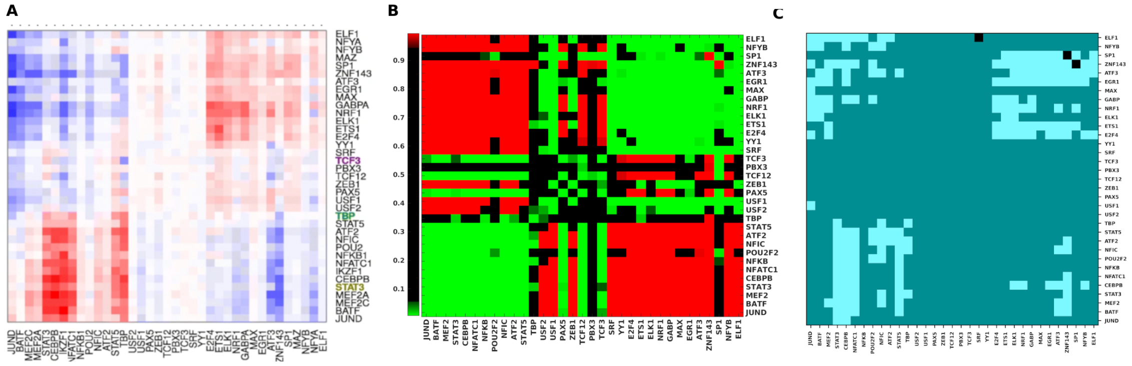

Figure A.3: (A)The heatmap from the earlier study by Ma et al. (2018)[15] ((reproduced under licence CC-BY-4.0)) showing attracting TF pairs in red and repelling pairs in blue. (B) The heatmap shows the attracting and repelling pairs in green and red respectively for common TFs used in both studies, using the method we proposed in methods section. (C) The heatmap shows a qualitative comparison of the two studies as follows: bright blue = both significant, in agreement; black = both significant, in disagreement (one showing attraction, the other repulsion); dark blue = one or both insignificant.

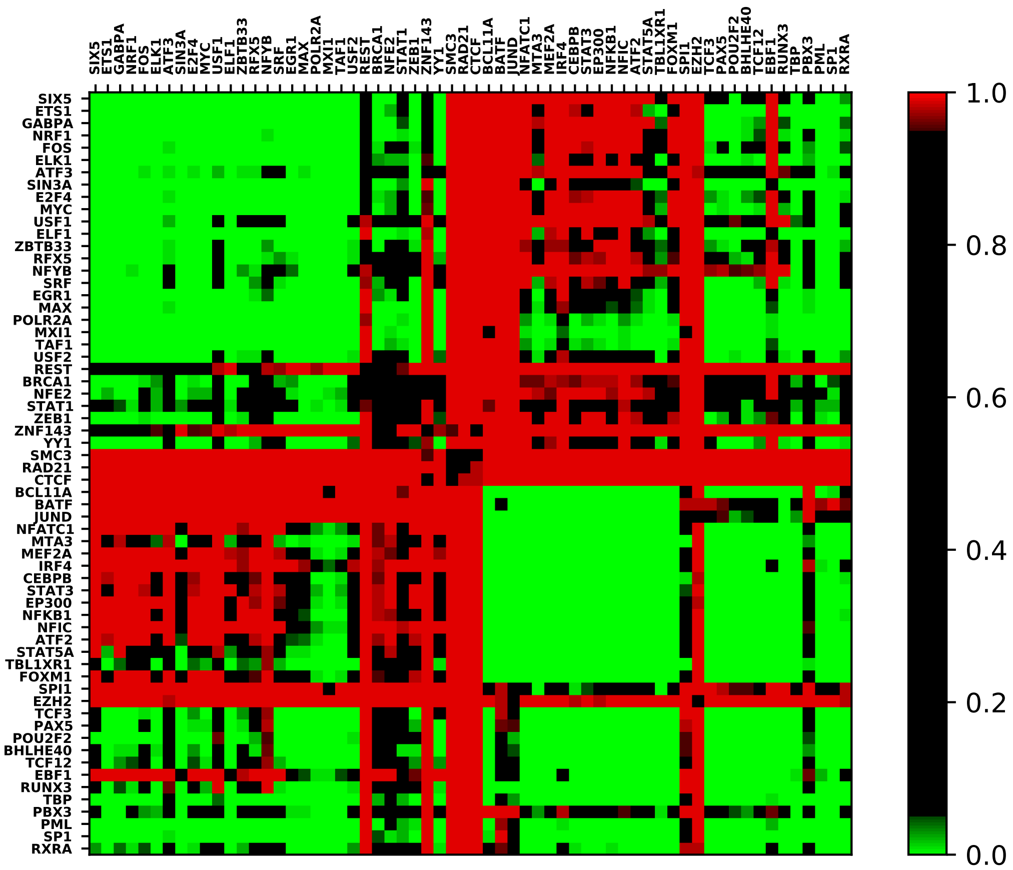

Figure A.4: The heatmap shows the co-occurrence pattern of TF pairs obtained using the Hi-C data used in the previous study by Ma *et al.* The order of TFs is same as used in figure2(A) and the co-occurrence is mostly similar to the pattern obtained using Pol II ChIA-PET data.

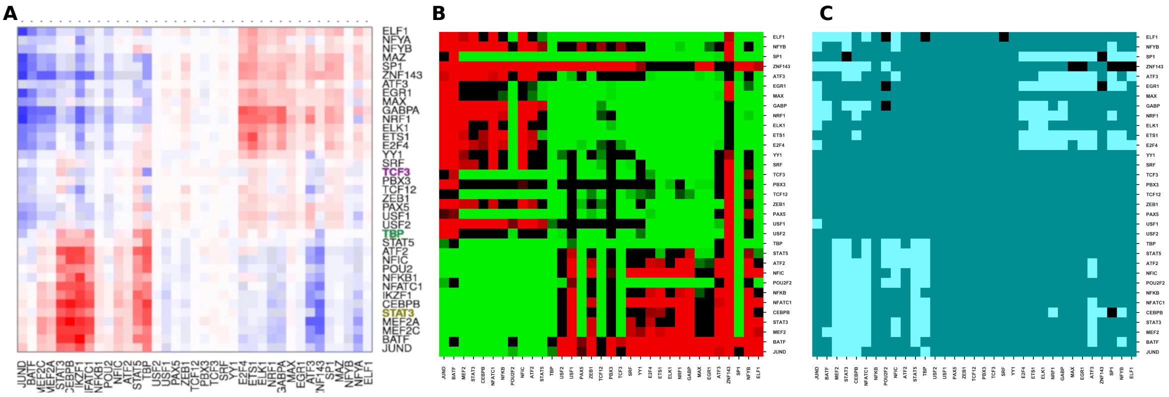

Figure A.5: (A)The heatmap from the earlier study by Ma et al. (2018)[15] ((reproduced under licence CC-BY-4.0)) showing attracting TF pairs in red and repelling pairs in blue. (B) The heatmap shows the attracting and repelling pairs in green and red respectively for common TFs used in both studies, using the method we proposed in methods section on Hi-C data. (C) The heatmap shows a qualitative comparison of the two studies as follows: bright blue = both significant, in agreement; black = both significant, in disagreement (one showing attraction, the other repulsion); dark blue = one or both insignificant.

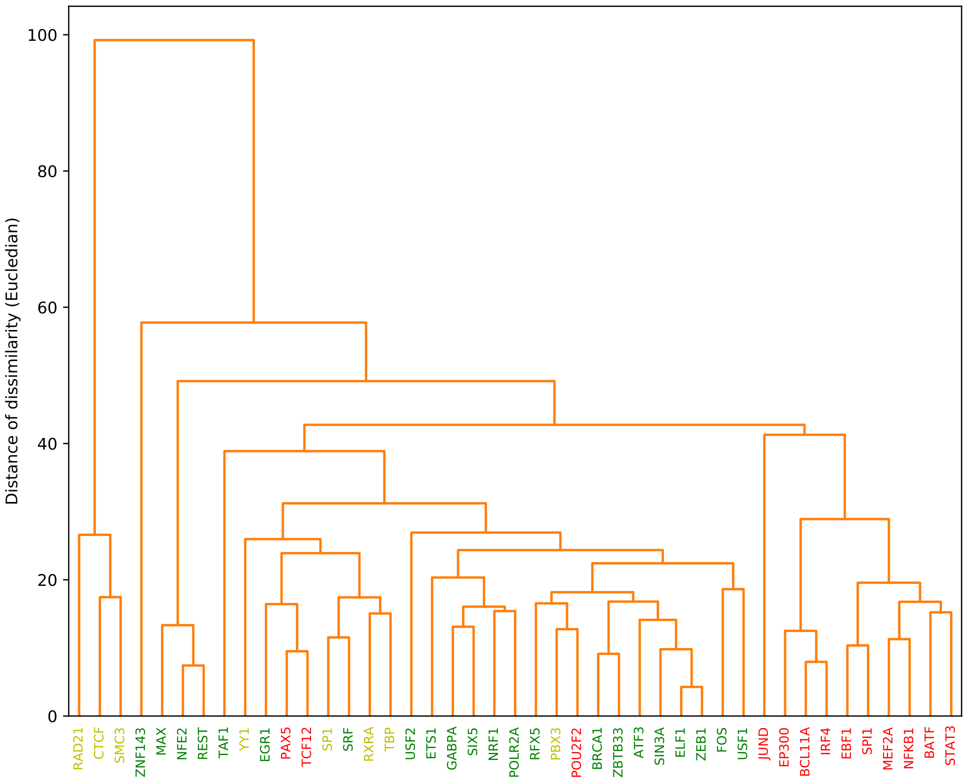

Figure A.6: Dendrogram shows similar grouping of TFs as in our study (Group 1 TFs in green, Group 2 TFs in red) from Encode study [25]

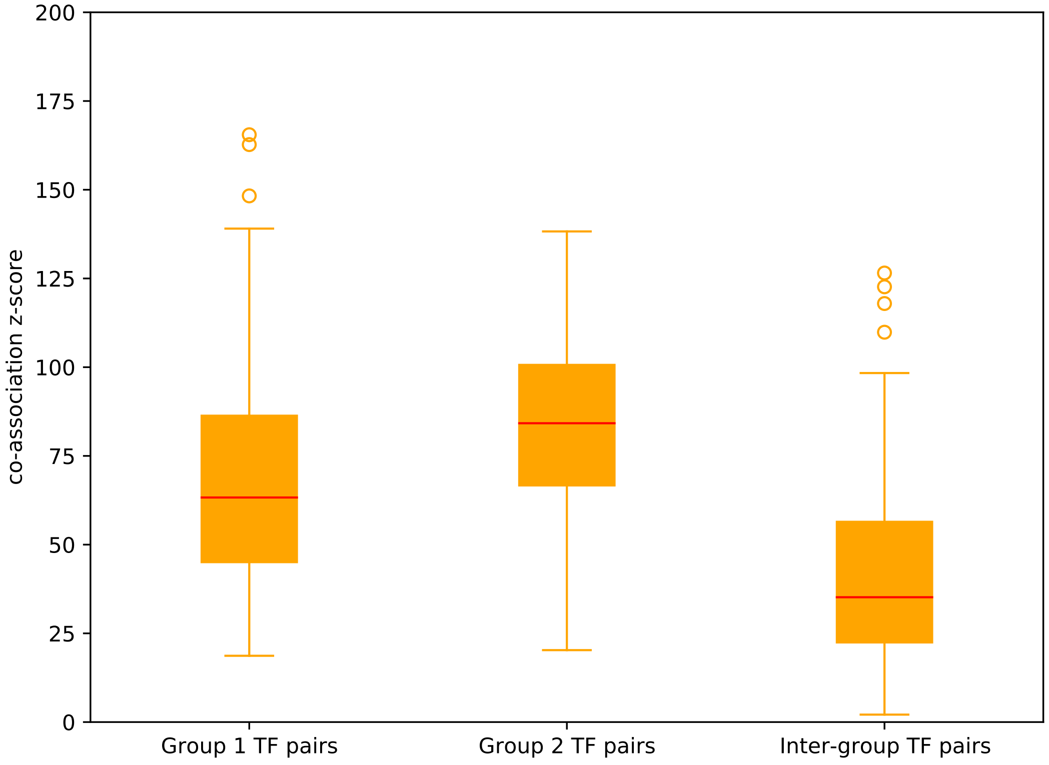

Figure A.7: The box plot shows significant difference with higher co-localization scores for TF pairs within a group to the inter-group TF pairs in the GM12878 cell line.

Co-occurrence of various histone marks

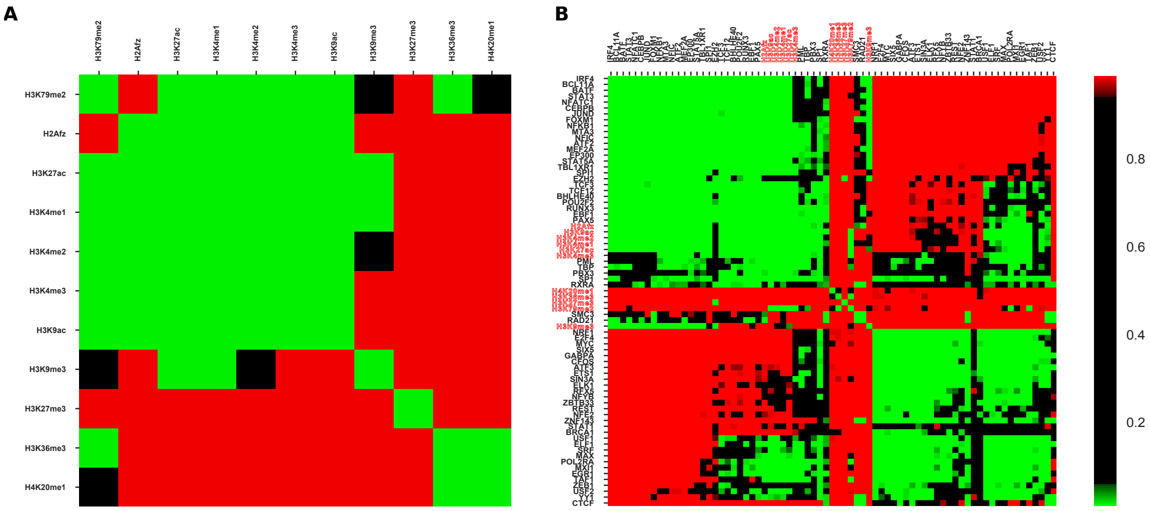

Figure A.8: (A) Clustered q-value heatmap similar to figure 2 for various histone marks. (B) shows the co-occurrence pattern for all the TFs along with the histone marks (labelled in red).

Co-occurrence pattern in A & B compartments of the genome

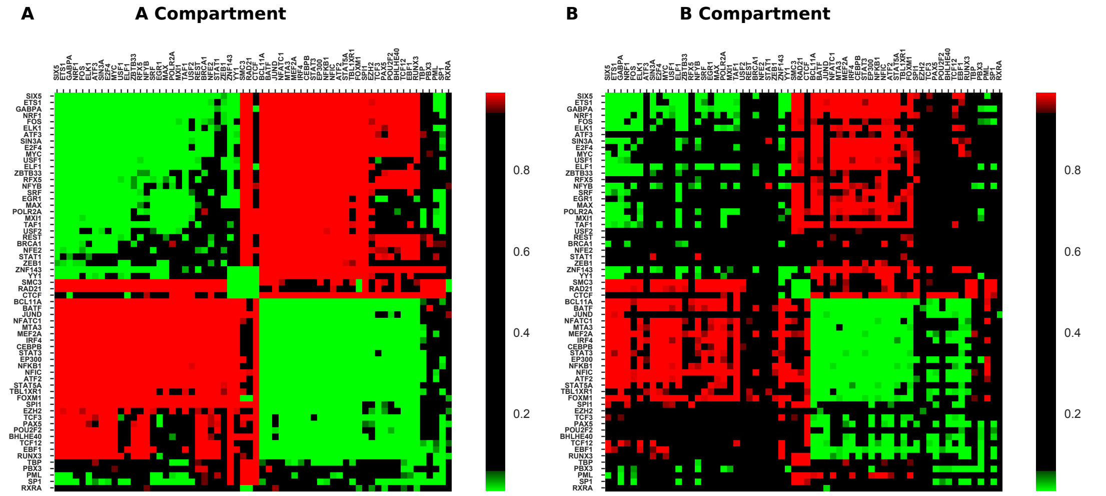

Figure A.9: (A) Shows q-value heatmap of co-occurrence of TF pairs in compartment A interactions of the genome and similarly (B) shows for compartment B interactions of genome of GM12878 cell line.

Co-occurrence of TFBS in HeLa-S3 cell line

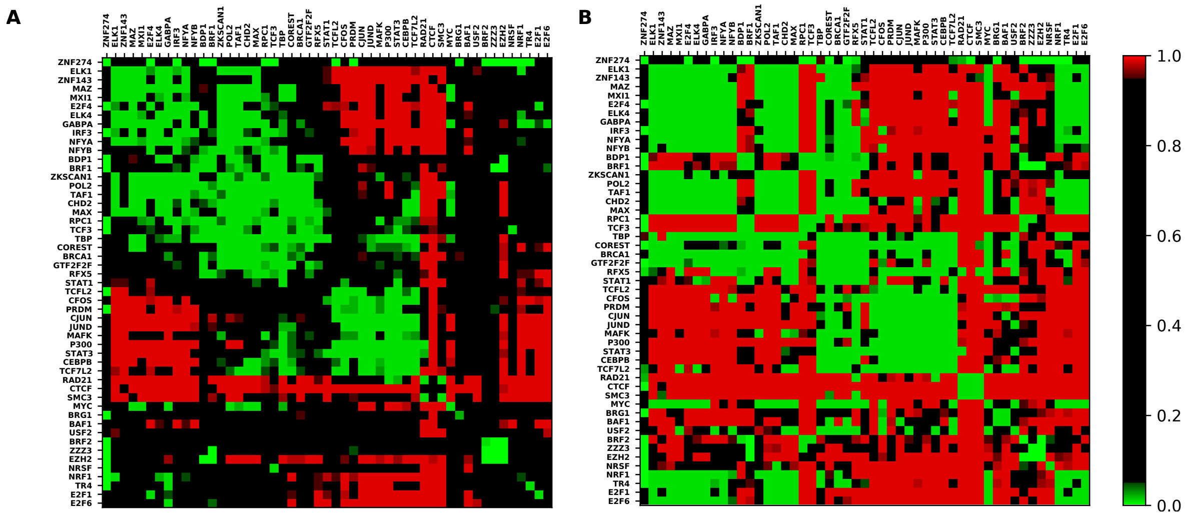

Figure A.10: (A) Shows the co-occurrence of TF pairs in spatial proximal regions. (B) shows the pattern in sequential contiguous regions

Comparison of pattern between cell lines

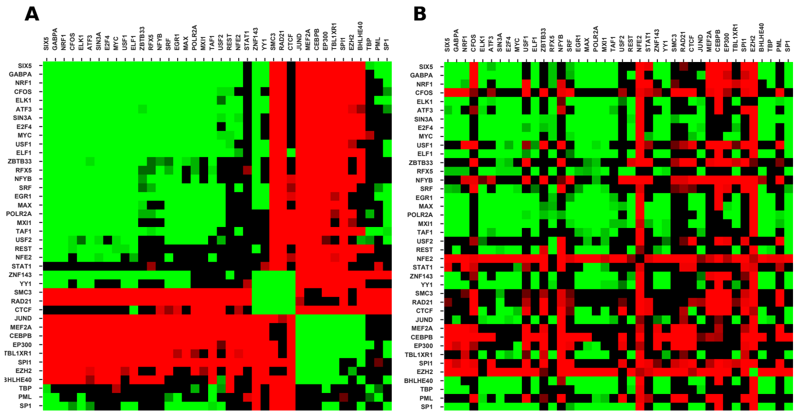

Figure A.11: A comparison of co-occurrence pattern in spatial proximal regions between GM12878 and K562 cell lines for the common factors present in both. (A) shows heatmap for GM12878 cell line, (B) shows heatmap for K562 cell line.

Co-occurrence pattern at motif level

Motif information is available many more transcription factors than the number of factors for which ChIP-seq data is available. But several of these TFs share similar motifs. We identified clusters of similar motifs using TOMTOM [23] and selected one TF representing the clusters, which was further used in the co-occurrence analysis. The Identified clusters motifs and their logos are shown in the file `https://figshare.com/s/5285cd308c2259ac5465`.

The following table gives motifs selected as an informative motif from each cluster.

Transcription factor	JASPAR Motif ID
Tbxt	MA0009.1
ELK1	MA0028.2
Gata1	MA0035.3
Gfi1	MA0038.1
FOXI1	MA0042.1
MAX	MA0058.3
NFYA	MA0060.3
RXRA::VDR	MA0074.1
ELK4	MA0076.2
Sox17	MA0078.1
SP1	MA0079.3
SPI1	MA0080.4
SRF	MA0083.3
ZNF143	MA0088.2
TEAD1	MA0090.1
ZEB1	MA0103.2
NFKB1	MA0105.4
TP53	MA0106.1
TBP	MA0108.1
ESR1	MA0112.3
NR3C1	MA0113.3
NFIC::TLX1	MA0119.1
Nkx3-1	MA0124.2
HINFP	MA0131.2

STAT1	MA0137.2
REST	MA0138.1
CTCF	MA0139.1
Sox2	MA0143.3
Tcfcp2l1	MA0145.1
Myc	MA0147.1
FOXA1	MA0148.3
NFATC2	MA0152.1
EBF1	MA0154.3
FOXO3	MA0157.2
EGR1	MA0162.3
CDX2	MA0465.1
DUX4	MA0468.1
E2F4	MA0470.1
ELF1	MA0473.1
Gfi1b	MA0483.1
HSF1	MA0486.2
JUND	MA0491.1
MAFK	MA0496.2
POU2F2	MA0507.1
PRDM1	MA0508.2
RFX5	MA0510.2
RUNX2	MA0511.2
Rxra	MA0512.2
Tcf12	MA0521.1
Esrra	MA0592.2
Hoxa9	MA0594.1
SREBF1	MA0595.1
FOXG1	MA0613.1
Mitf	MA0620.1
mix-a	MA0621.1
BARHL2	MA0635.1
BHLHE41	MA0636.1
ETV6	MA0645.1
GRHL1	MA0647.1
IRF9	MA0653.1
NFIA	MA0670.1
NKX2-3	MA0672.1
ONECUT1	MA0679.1
PAX7	MA0680.1
POU4F2	MA0683.1
SP4	MA0685.1
ZBTB7B	MA0694.1
OTX2	MA0712.1
GLIS1	MA0735.1
GLIS3	MA0737.1
Hic1	MA0739.1
SCRT2	MA0744.1
E2F7	MA0758.1
Tcf7	MA0769.1
MEF2D	MA0773.1
MEIS3	MA0775.1

PAX1	MA0779.1
PAX9	MA0781.1
POU3F4	MA0789.1
SMAD3	MA0795.1
TGIF1	MA0796.1
TFAP2C(var.3)	MA0815.1
ATF7	MA0834.1
NFE2	MA0841.1
TEF	MA0843.1
Rarb	MA0857.1
Rarg(var.2)	MA0860.1
E2F2	MA0864.1
SOX4	MA0867.1
Sox11	MA0869.1
TFAP2A(var.3)	MA0872.1
HOXC13	MA0907.1
SIX1	MA1118.1

Table A.3: The selected motifs after clustering for similar motifs using TOMTOM

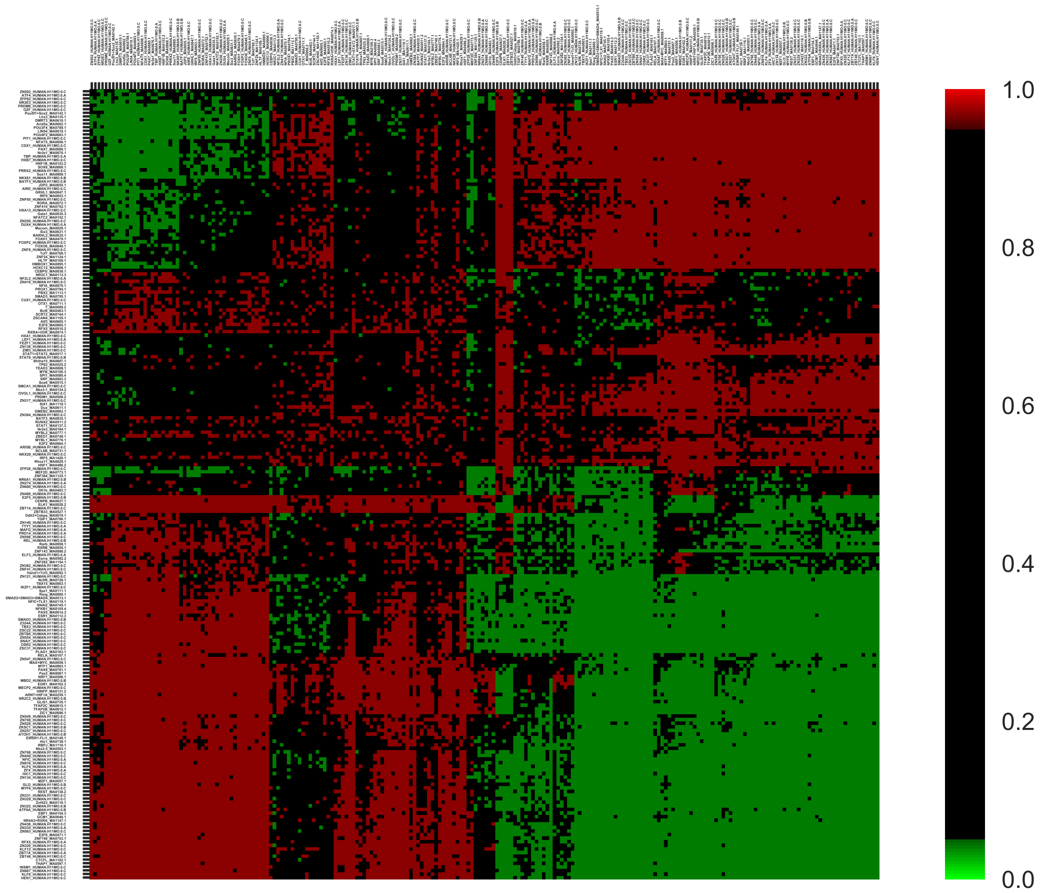

Figure A.12: Heatmap shows co-occurrence pattern with the high informative motifs of clustered motif models from Vierstra et al. (2020) [35] for GM12878 cell line.

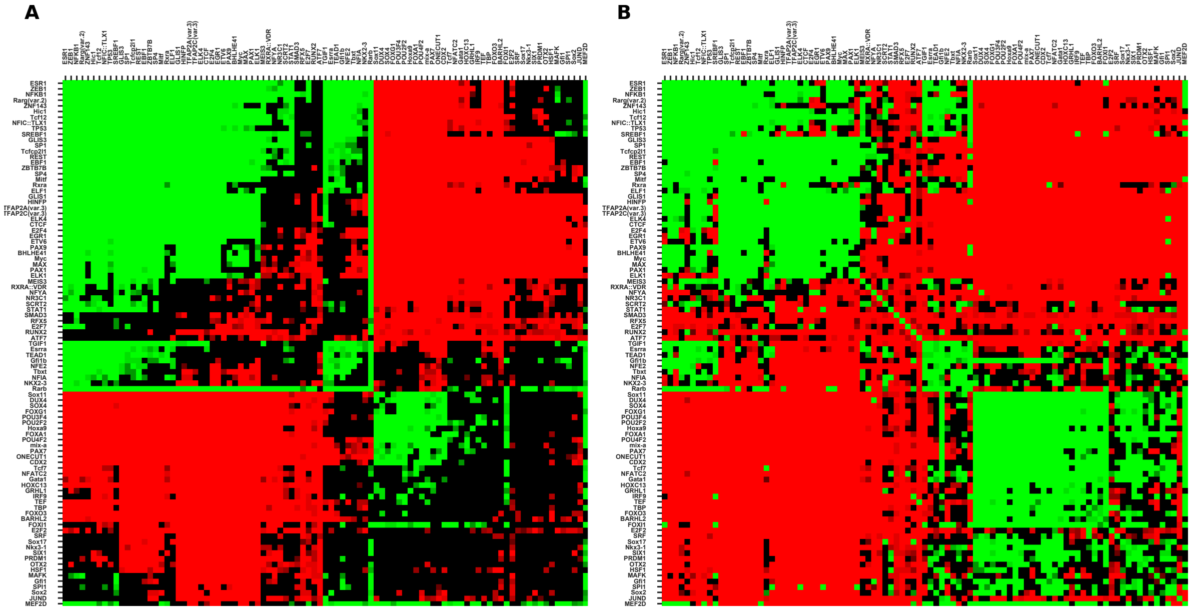

Figure A.13: (A) The co-occurrence of TF motifs sites in spatial proximal regions and (B) in sequential contiguous regions of GM12878 cell line

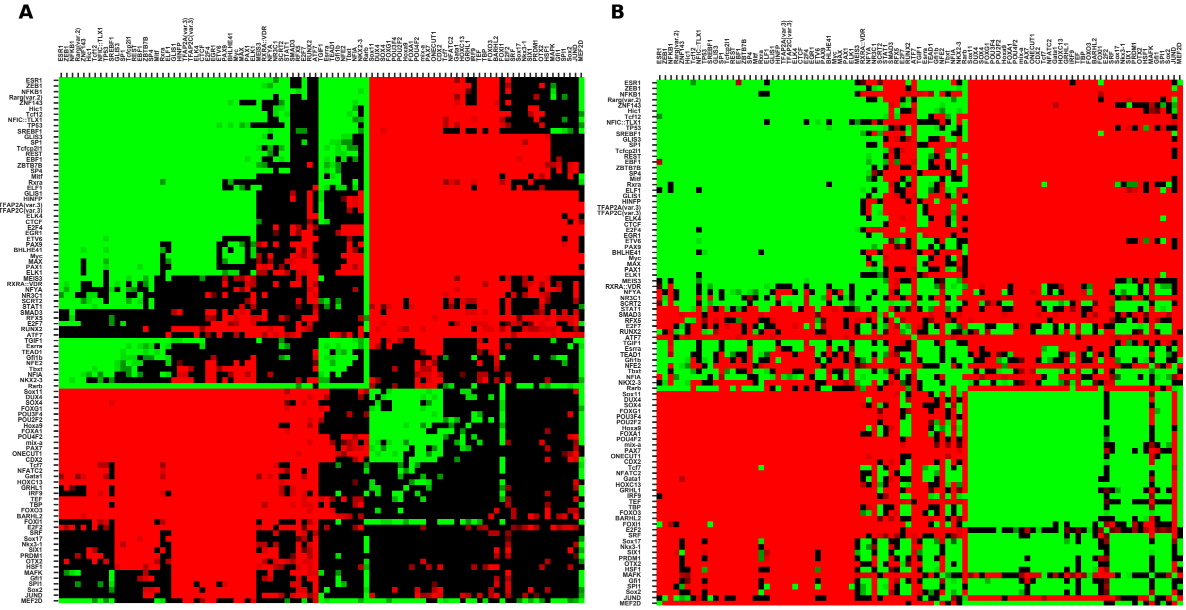

Figure A.14: (A) The co-occurrence of TF motifs sites in spatial proximal regions and (B) in sequential contiguous regions of k562 cell line

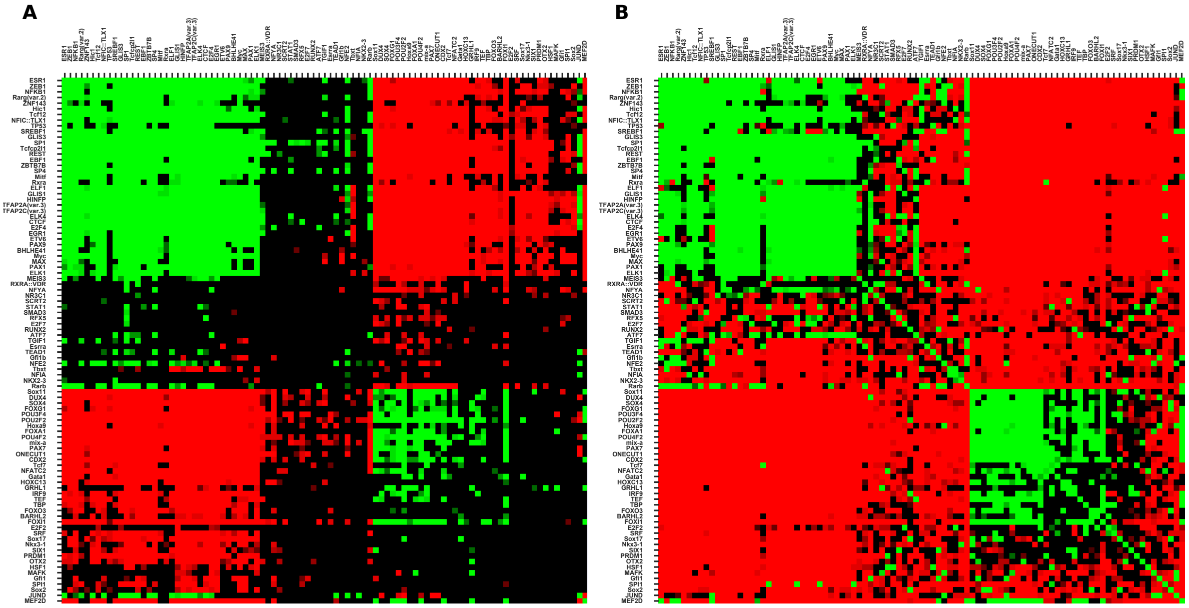

Figure A.15: (A) The co-occurrence of TF motifs sites in spatial proximal regions and (B) in sequential contiguous regions of HeLa-S3 cell line

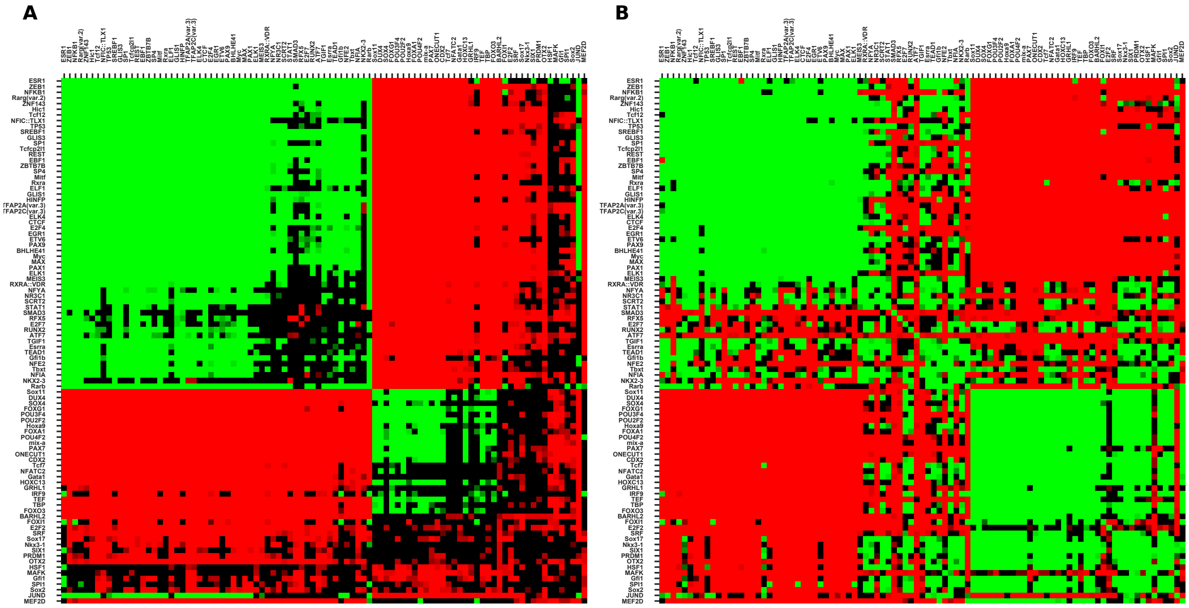

Figure A.16: (A) The co-occurrence of TF motifs sites in spatial proximal regions and (B) in sequential contiguous regions of MCF7 cell line

Comparison of all four types of co-occurrence patterns

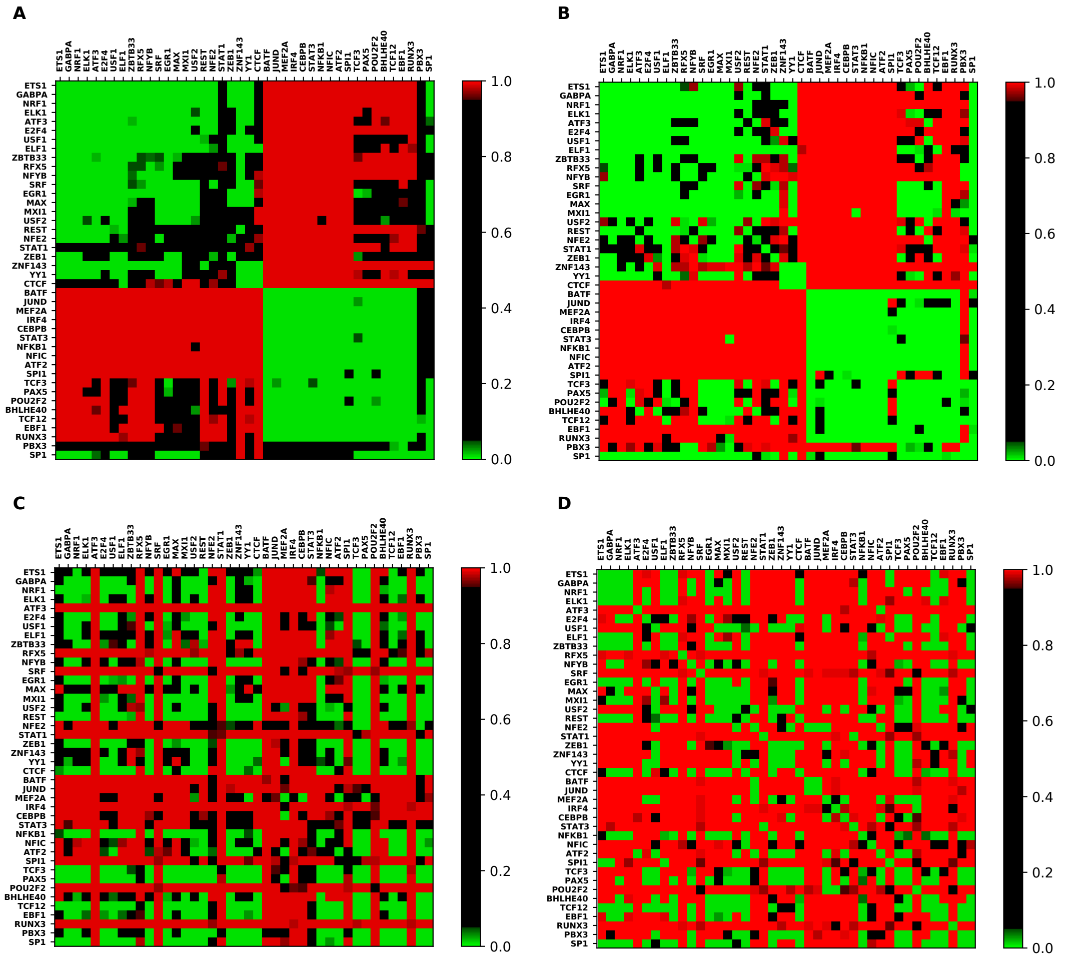

Figure A.17: The figure shows all the four different q-value heatmaps of GM12878 cell line. (A) using TF chip-seq peaks in spatial proximal regions, (B) using TF chip-seq peaks in sequential contiguous regions, (C) using TF motif sites in spatial proximal regions, and (D) using TF motif sites in sequential contiguous regions.

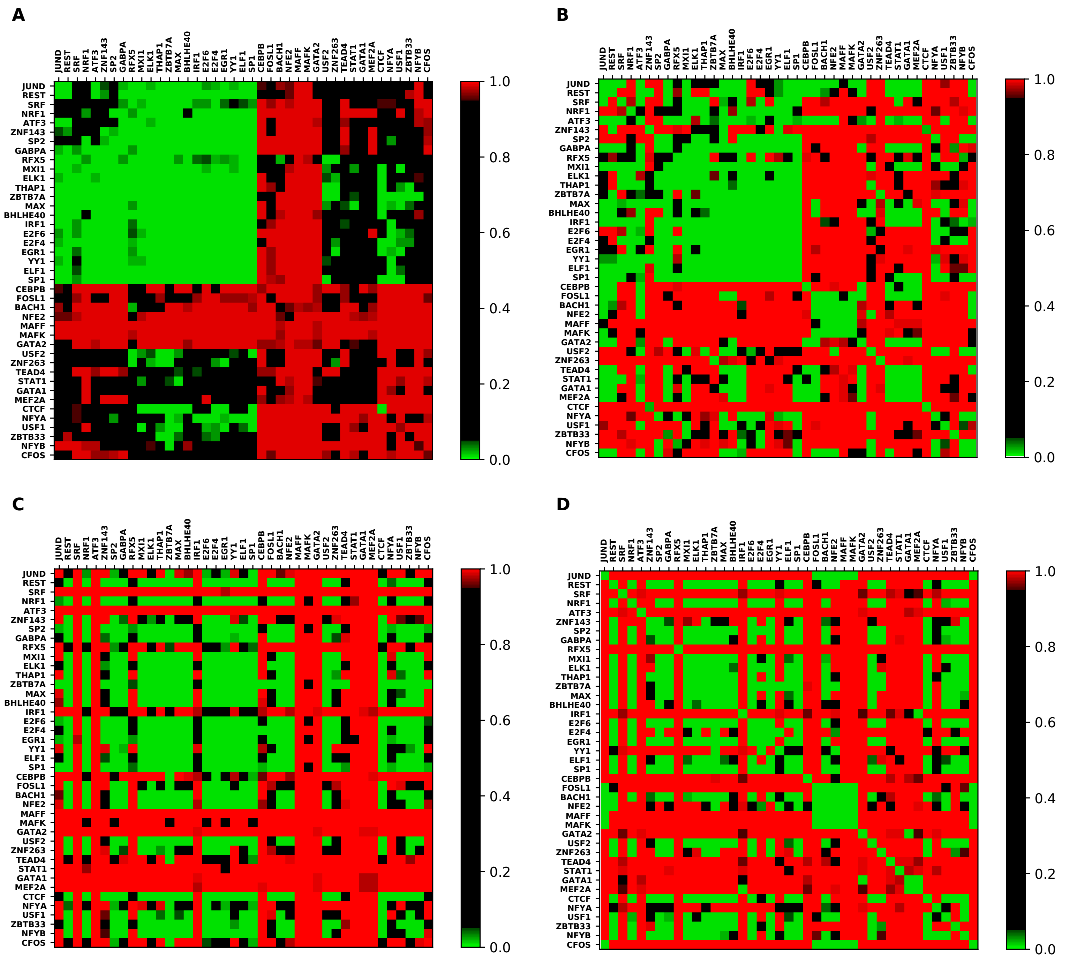

Figure A.18: The figure shows all the four different q-value heatmaps of K562 cell line. (A) using TF chip-seq peaks in spatial proximal regions, (B) using TF chip-seq peaks in sequential contiguous regions, (C) using TF motif sites in spatial proximal regions, and (D) using TF motif sites in sequential contiguous regions.

Functional difference between the groups of TFs

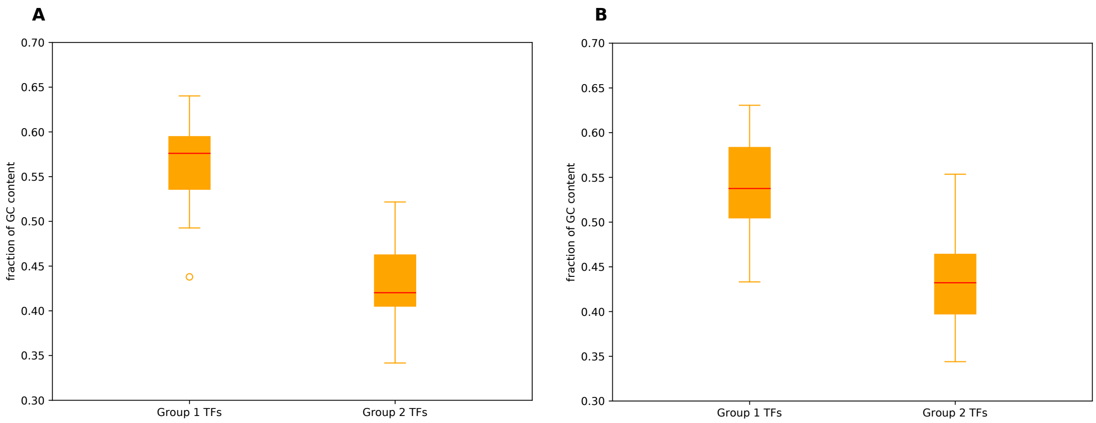

Figure A.19: Box plots show the GC composition difference among Group 1 and Group 2 TF chip-seq peak sequences in (A) GM12878 and (B) K562 cell line

Motif strength analysis

Transcription factor	JASPAR motif ID
ETS1	MA0098.3
GABPA	MA0062.1
NRF1	MA0506.1
ELK1	MA0028.2
ATF3	MA0605.2
E2F4	MA0470.2
USF1	MA0093.2
ELF1	MA0473.2
ZBTB33	MA0527.1
RFX5	MA0510.2
NFYB	MA0502.2
SRF	MA0083.1
EGR1	MA0162.3
MAX	MA0058.1
MXI1	MA1108.1
USF2	MA0526.1
REST	MA0138.2
NFE2	MA0841.1
STAT1	MA0137.3
ZEB1	MA0103.2
ZNF143	MA0088.2
YY1	MA0095.2
CTCF	MA0139.1
BATF	MA1634.1
JUND	MA0491.1
MEF2A	MA0052.3
IRF4	MA1419.1
CEBPB	MA0466.2
STAT3	MA0144.2
NFKB1	MA0105.2
NFIC	MA0161.2
ATF2	MA1632.1

SPI1	MA0080.4
TCF3	MA0522.2
PAX5	MA0014.2
POU2F2	MA0507.1
BHLHE40	MA0464.2
TCF12	MA1648.1
EBF1	MA0154.3
RUNX3	MA0684.1
PBX3	MA1114.1
SP1	MA0079.4

Table A.4: This table gives the JASPAR motif ids for the TF used in the motif strength analysis in Figure 8

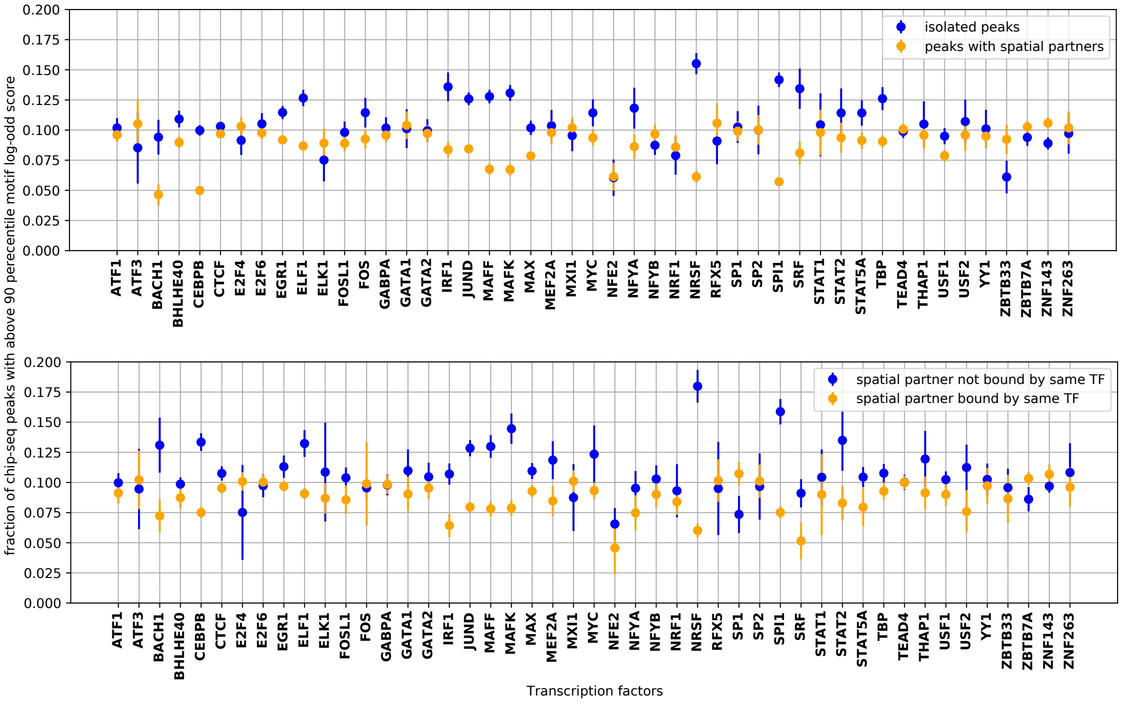

Figure A.20: (A) Spatially isolated ChIP-seq peaks have a higher fraction of strong motifs than peaks with spatial clusters. (B) Even among the peaks with spatial partners, the peaks with the partner region not bound by same TF show higher fraction of strong motifs than bound by the same TF.

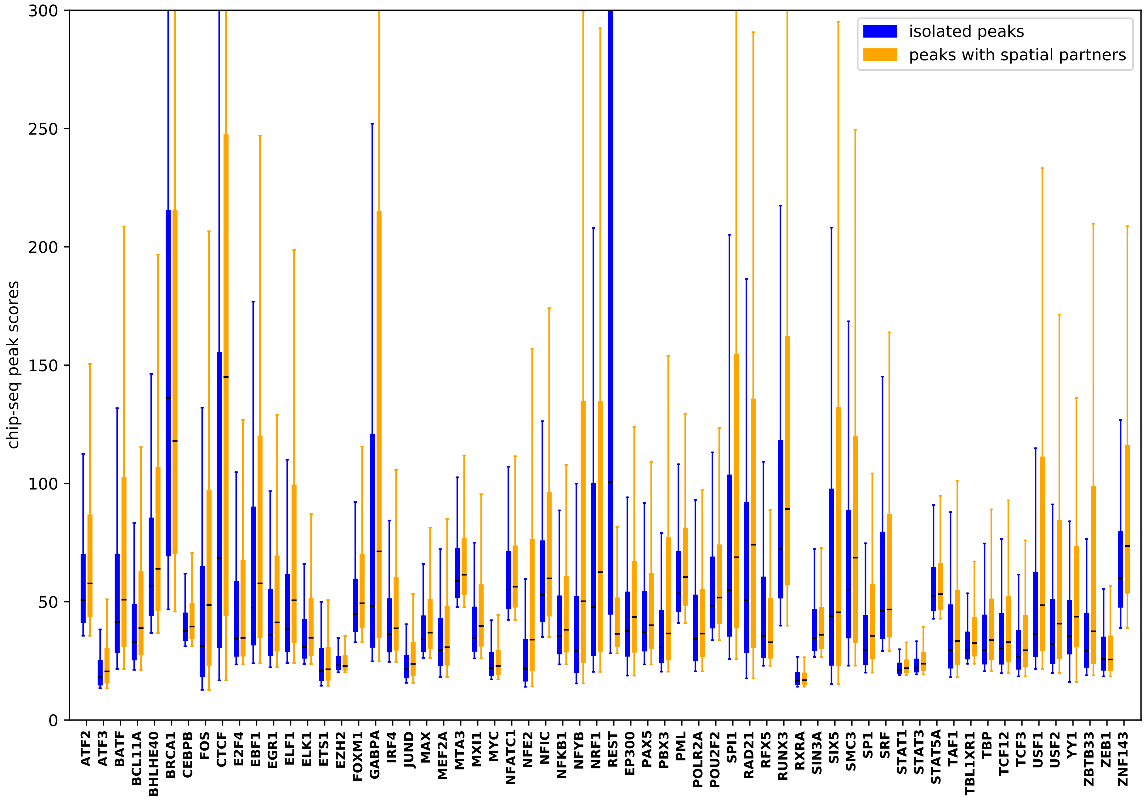

Figure A.21: The box plots show the distribution of chip-seq peak strength scores of peaks present on spatially isolated and spatially interacting chromatin regions.

Target gene analysis

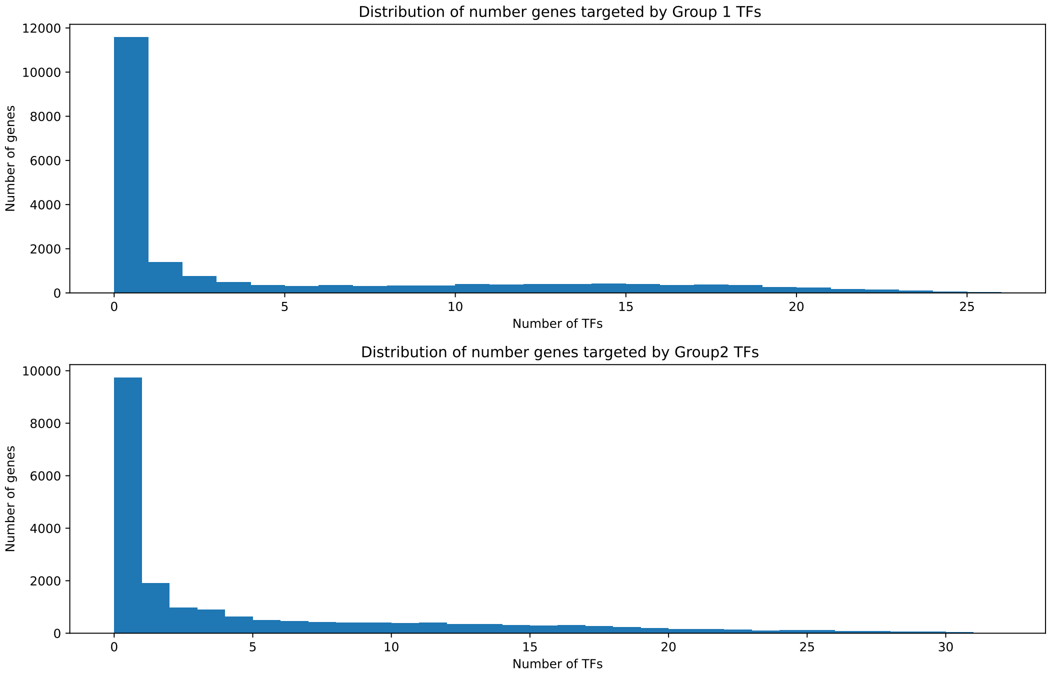

Figure A.22: Distribution of number of putative TF regulators per gene in GM12878 cell line.

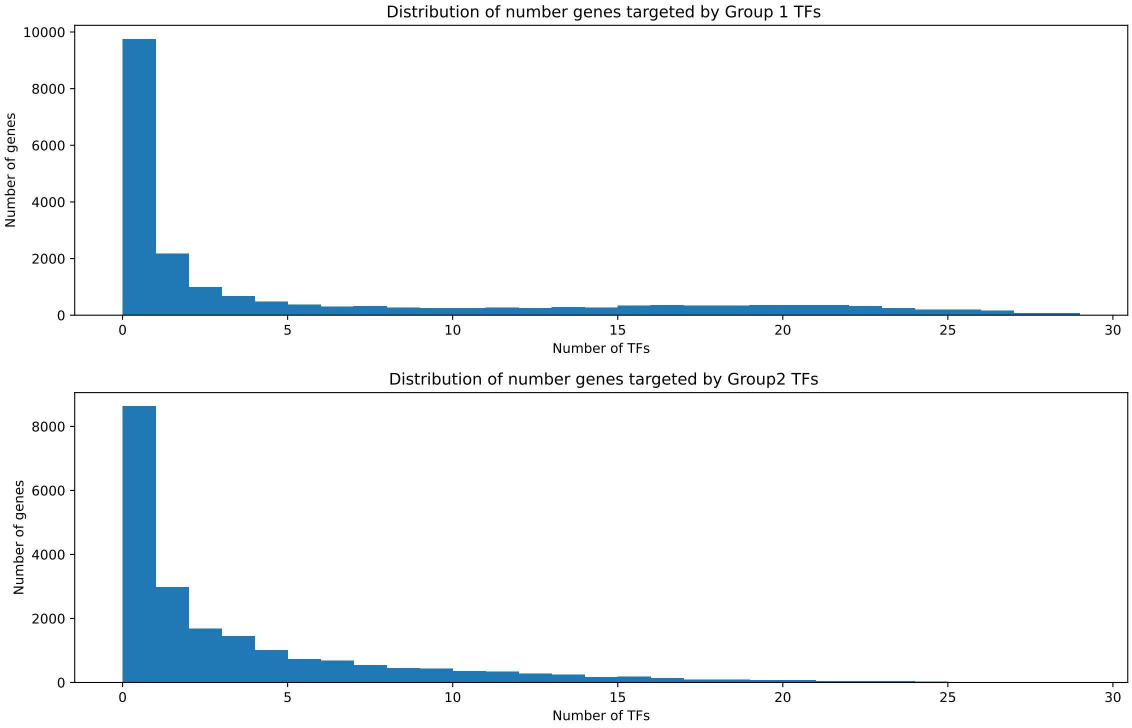

Figure A.23: Distribution of number of putative TF regulators per gene in K562 cell line.

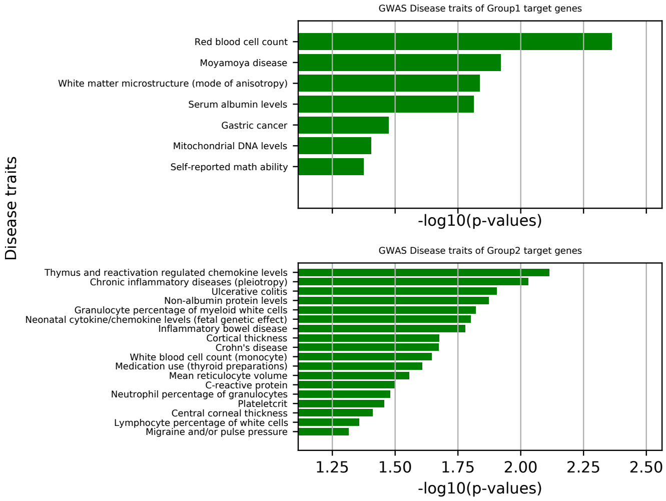

Figure A.24: The enriched GWAS disease traits of the target genes of Group 1 and Group 2 TFs

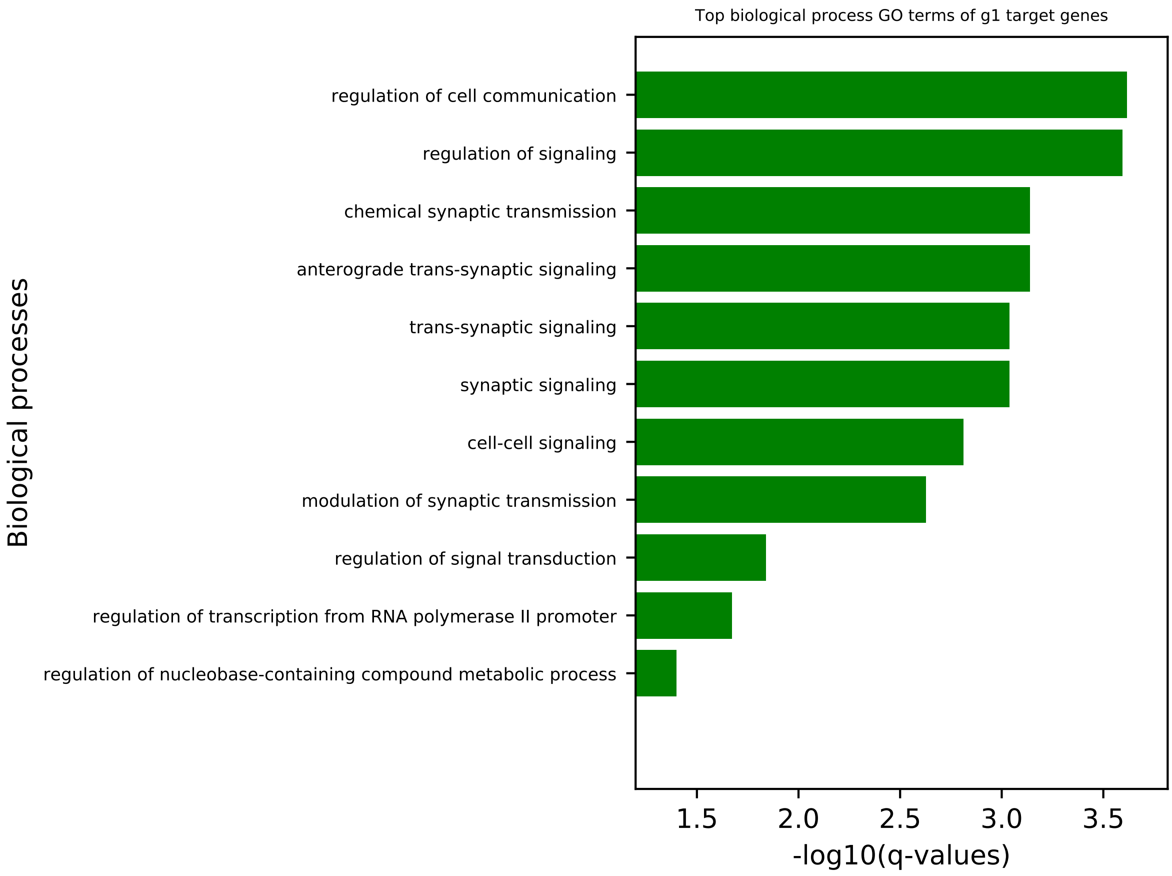

Figure A.25: The enriched GO biological processes for the Group 1 TFs of the K562 cell line

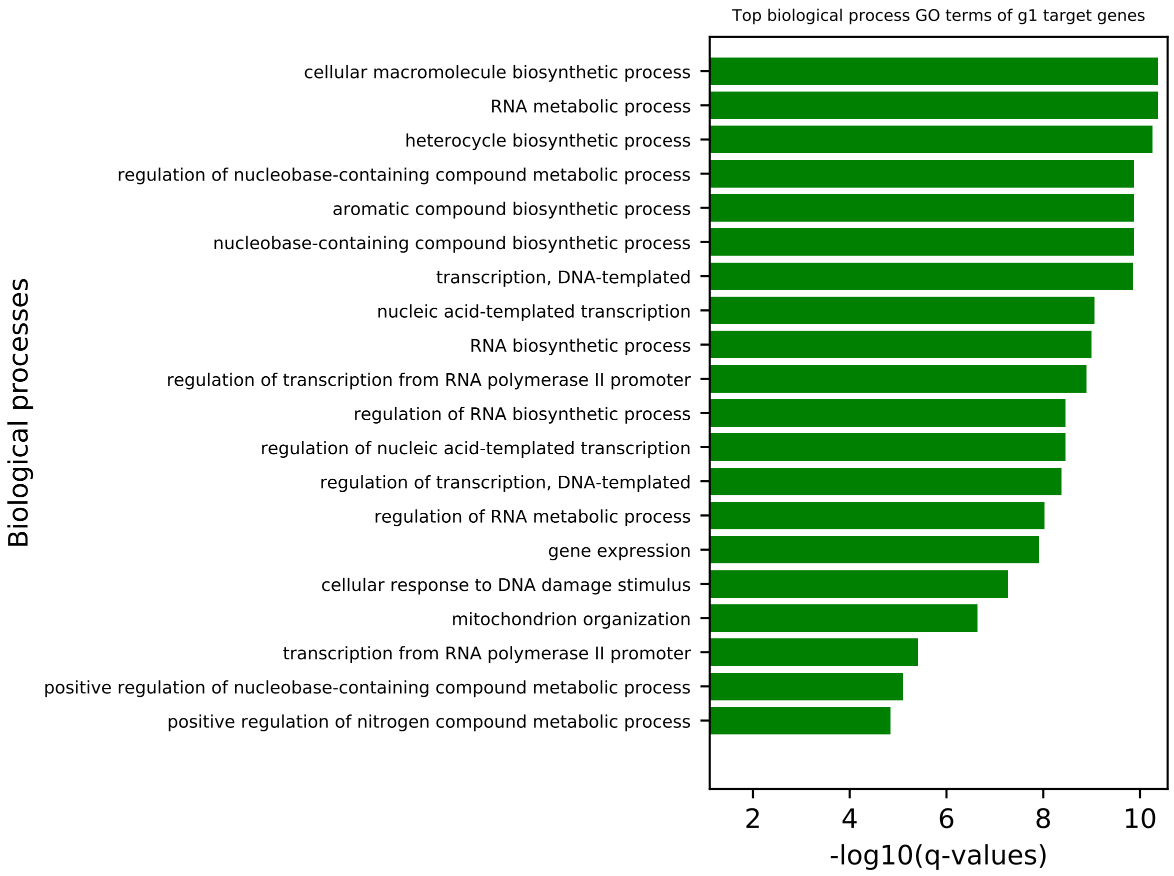

Figure A.26: The enriched GO biological processes for the Group 1 TFs of the HeLa-S3 cell line
